# Strain Variation in the Transcriptome of the Dengue Fever Vector, *Aedes aegypti*

**DOI:** 10.1534/g3.111.001107

**Published:** 2012-01-01

**Authors:** Mariangela Bonizzoni, W. Augustine Dunn, Corey L. Campbell, Ken E. Olson, Osvaldo Marinotti, Anthony A. James

**Affiliations:** * Program in Public Health; † Department of Molecular Biology and Biochemistry, and; ‡ Institute for Genomics and Bioinformatics, University of California, Irvine, California 92697; § Department of Biochemistry and Molecular Biology; ** Department of Microbiology, Immunology and Pathology, Colorado State University, Fort Collins, Colorado 80523; †† Department of Microbiology and Molecular Genetics, University of California, California, Irvine 92697

**Keywords:** *Aedes aegypti*, strain variation, bloodmeal, RNA-seq

## Abstract

Studies of transcriptome dynamics provide a basis for understanding functional elements of the genome and the complexity of gene regulation. The dengue vector mosquito, *Aedes aegypti*, exhibits great adaptability to diverse ecological conditions, is phenotypically polymorphic, and shows variation in vectorial capacity to arboviruses. Previous genome sequencing showed richness in repetitive DNA and transposable elements that can contribute to genome plasticity. Population genetic studies revealed a varying degree of worldwide genetic polymorphism. However, the extent of functional genetic polymorphism across strains is unknown. The transcriptomes of three *Ae. aegypti* strains, Chetumal (CTM), Rexville D-Puerto Rico (Rex-D) and Liverpool (LVP), were compared. CTM is more susceptible than Rex- D to infection by dengue virus serotype 2. A total of 4188 transcripts exhibit either no or small variation (<2-fold) among sugar-fed samples of the three strains and between sugar- and blood-fed samples within each strain, corresponding most likely to genes encoding products necessary for vital functions. Transcripts enriched in blood-fed mosquitoes encode proteins associated with catalytic activities, molecular transport, metabolism of lipids, carbohydrates and amino acids, and functions related to blood digestion and the progression of the gonotropic cycle. Significant qualitative and quantitative differences were found in individual transcripts among strains including differential representation of paralogous gene products. The majority of immunity-associated transcripts decreased in accumulation after a bloodmeal and the results are discussed in relation to the different susceptibility of CTM and Rex-D mosquitoes to DENV2 infection.

*Aedes aegypti* (Diptera, Culicidae) is the primary vector for dengue (DEN), yellow fever, and Chikungunya viruses throughout most tropical and subtropical areas of the world (Charrel *et al.* 2007; Gubler 2002). In addition to its significance as a public health concern, ease of laboratory breeding and maintenance makes it a good vector mosquito model system (Clemons *et al.* 2010; Kuno 2010). Importantly, a key component of a model organism is the ability to use information gathered from transcriptomics from one strain to make inferences about other strains within the species. This information then can be used in studies that test possible interactions among genotypic variation, transcriptional regulation, and environmental factors (Wagner 2011).

This mosquito species is phenotypically polymorphic, has great adaptability to diverse ecological conditions, and shows variation in vectorial capacity for arboviruses (Bennett *et al.* 2002; Black *et al.* 2002; Kuno 2010). Genetic polymorphism among geographically distinct *Ae. aegypti* populations is documented (Urdaneta-Marquez and Failloux 2011); however, the extent of genome sequence polymorphism and its effects on transcriptional activity are not known.

The transcriptional profiles of three *Ae. aegypti* strains, Liverpool (LVP), Chetumal (CTM), and Rexville D Puerto Rico (Rex-D), were investigated. LVP originated in West Africa in the 1930s, and its genome is sequenced (Nene *et al.* 2007), CTM was derived from the Yucatan Peninsula in Mexico in the early 2000s (Bennett *et al.* 2002; Gubler *et al.* 1985; Richardson *et al.* 2006), whereas Rex-D was established from mosquitoes captured in Puerto Rico in the early 1990s (Miller and Mitchell 1991). CTM supports a faster and more intense dissemination of dengue virus serotype 2 (DENV2) than Rex-D (Bennett *et al.* 2002; Salazar *et al.* 2007). Our data show significant differences in transcript accumulation among strains, and the results may account for the differing susceptibility of CTM and Rex-D mosquitoes to virus infection.

## Material and Methods

### *Aedes aegypti* strains

LVP, CTM, and Rex-D mosquitoes were reared under identical laboratory conditions to prevent the effects of environmental factors on transcription. Female and male mosquitoes were kept together in cages with unlimited access to sugar (raisins) and water until blood feeding. Three- to five day-old female mosquitoes were allowed to feed on anesthetized mice. Blood-fed females were transferred to another cage, kept with access to water and sugar, and whole-animal samples harvested at 5, 8, 12, 24, or 72 hr post-bloodmeal (hPBM) and stored at −80°. Blood-feedings occurred between 8 and 10 am to avoid differences in expression profiles attributable to circadian rhythms (Ptitsyn *et al.* 2011).

### Weight comparisons of sugar and blood-fed mosquitoes

Three-day old females were separated into pools of 20 mosquitoes each, denied access to water for 4 hr, immobilized by CO_2_, and weighed. Mosquitoes then were given access to water and sucrose until 4 hr before blood-feeding. At 24 hr after the first weight measurement, six pools of each strain were allowed to feed on anesthetized mice for 15 min. Immediately after blood-feeding, each pool was immobilized by CO_2_ and reweighed. Two additional weight measurements were recorded at 30-min intervals to account for diuresis (Stobbart 1977). The differences in weight before and after blood-feeding and among the three strains were analyzed by a *t*-test, assuming equal variance.

### RNA-seq library preparation and sequencing

Total RNA was extracted with TRIZOL (Invitrogen) from pools of three female mosquitoes either kept on a sugar diet (S) or 5 hr after blood-feeding (B), The quality of the RNA was checked on an Agilent 2100 Bioanalyzer, and two samples each of S and B mosquitoes were pooled for RNA-seq library preparation. Libraries were prepared following the standard Illumina protocol (http://www.illumina.com/products/mrna_seq_8_sample_prep_kit.ilmn) and sequenced with 40-bp single reads at the Expression Analysis Core at the UC Davis Genome Center (http://genomecenter.ucdavis.edu/expression_analysis/). Libraries were run at a nucleic acid concentration of 4 to 5 pM.

### Reverse transcription gene amplification

Total RNA was extracted using TRIZOL (Invitrogen) from pools of 300 embryos collected 12 hr after egg deposition, 30 3rd instar larvae, 30 pupae, or five adults. Adult samples included 3- to 5-day-old male or female mosquitoes kept on a sugar diet and 3- to 5-day-old females collected 5, 24 and 72 hPBM. Total RNA from six adult samples of each of the time points was pooled in equal amounts to have a representation of 30 individuals as in the case of larvae and pupae. After DNAse I treatment (Ambion), 800 ng of RNA were used for cDNA synthesis with Superscript III (Invitrogen) and random oligonucleotide primers. Gene amplification reactions were performed with the use of 2 μl of cDNA, 0.4 μM of each transcript-specific primer (supporting information, Table S1), 0.2 nM each dNTP, 2 mM MgSO_4_, 1X buffer, and 1 Unit of Platinum *Taq* DNA Polymerase (Invitrogen). Amplification conditions were 94° for 2 min followed by 25 to 40 cycles of 94° for 15 sec, 45 to 65° for 30 to 45 sec, 68° for 30 sec, and a final step at 68° for 6 min (Table S1). Amplification products were resolved in a 2% agarose gel and stained with GelRed (Phenix Research).

### Quantitative real-time gene amplification

Total RNA was extracted by TRIZOL (Invitrogen) from pools of eight females either kept exclusively on a sugar diet or eight females collected at 5, 8, 12, and 24 hPBM. After DNAse I (Invitrogen) treatment, 10 µg of RNA were used for cDNA synthesis with Superscript III (Invitrogen) and random primers. Real-time quantitative gene amplification reactions for 13 selected transcripts were run and analyzed as described previously (Bonizzoni *et al.* 2011).

### RNA-seq data analyses

RNA-seq data for the CTM and Rex-D strains are deposited at the NCBI Gene Expression Omnibus under accession number GSE32074. RNA-seq data for the LVP strain are available under the accession number GSE24872 (Bonizzoni *et al.* 2011). Sequence reads were mapped to the LVP reference genome with Bowtie (Langmead *et al.* 2009), allowing a maximum of two mismatches and with the –m option, which returns only reads with a single best match in the genome (Bonizzoni *et al.* 2011).

Differential transcript accumulation levels among conditions were assessed by the likelihood ratio test implemented in DEGseq (Wang *et al.* 2010), after accounting for the different total gene counts of each library, and at a *P* value of 0.001 with a false discovery rate of 0.1% (Benjamini and Hochberg 1995). Use of the modifier “significant” in the following text implies that accumulation values met the statistical criteria.

The reference file containing transcript annotation information and used in DEGseq was generated by converting a GTF annotation file obtained through http://metazoa.ensembl.org/ to the format (refflat) accepted by DEGseq and represents gene-build AaegL1.2. Function parent attribution of the sequenced transcripts is based on the *Ae. aegypti* database AegyXcel (http://exon.niaid.nih.gov/transcriptome.html#aegyxcel). Transcripts accumulated differentially among samples were visualized in the *Ae. aegypti* protein network (Guo *et al.* 2010) with the use of Cytoscape_v2.7.0 (http://www.cytoscape.org/). Metabolic pathways were analyzed by LinkinPath (Ingsriswang *et al.* 2011), which accepts a maximum of 5000 sequences per session and the color of each group of sequences is assigned automatically.

## Results

### RNA-seq mapping summary

Transcriptional profiles of LVP, CTM, and Rex-D strains before and after a bloodmeal were compared. RNA-seq libraries were generated with the use of RNA extracted from females collected 3 to 5 days after eclosion and kept either on a sugar diet (S) or harvested 5 hPBM (B) (Table 1). Two RNA-seq libraries were constructed for each RNA sample. The reproducibility of the parallel libraries was verified (Figure S1), and the data from the two technical replicates were merged for further analyses. Reads mapping to multiple genomic locations were discarded during Bowtie alignment. As a consequence, the accumulation levels of transcripts encoded by highly conserved gene families are under-estimated. This bias is expected to affect all samples equally; therefore, if the absolute accumulation level of a transcript is underestimated, differences across conditions still can be calculated.

**Table 1:** Experimental design and mapping summary

		Blood-Induced Changes*^a^*	
Strain		3-5 Days PE, S	5 hr PBM
LVP	N. reads*^b^*	7,576,184	15,539,328
	N. transcripts*^c^*	13,193 (70.33%)	13,668 (72.86%)
CTM	N. reads*^b^*	31,975,808	13,350,049
	N. transcripts*^c^*	13,153 (70.11%)	13,514 (72.04%)
Rex-D	N. reads*^b^*	11,616,156	13,824,315
	N. transcripts*^c^*	13,587 (72.43%)	13,581 (72.39%)

LVP, Liverpool CTM, Chetumal; Rex-D, Rexville D-Puerto Rico; PBM, post-bloodmeal; PE, posteclosion.

^a^ Blood-induced changes involved comparison between female mosquitoes 3-5 days post eclosion (PE) exclusively kept on a sugar diet (S) and blood-fed 3-5 day old females sampled 5 hPBM.

^b^ Total number of reads sequenced.

^c^ Total number (and percentage) of transcripts detected of the 18,760 *Ae. aegypti* transcripts annotated (gene build AaegL1.2).

RNA-seq reads mapped uniquely in the mosquito genome revealed that approximately 70% of the 18,760 *Ae. aegypti* transcripts (gene build AaegL1.2) are expressed in adult CTM, Rex-D, and LVP females (Table 1). The number of transcripts is not statistically different among strains, indicating that transcript detection is not biased by the number of sequences obtained *per* tested condition or by sequence polymorphism.

### Constitutively accumulated transcripts

**Figure 1:**
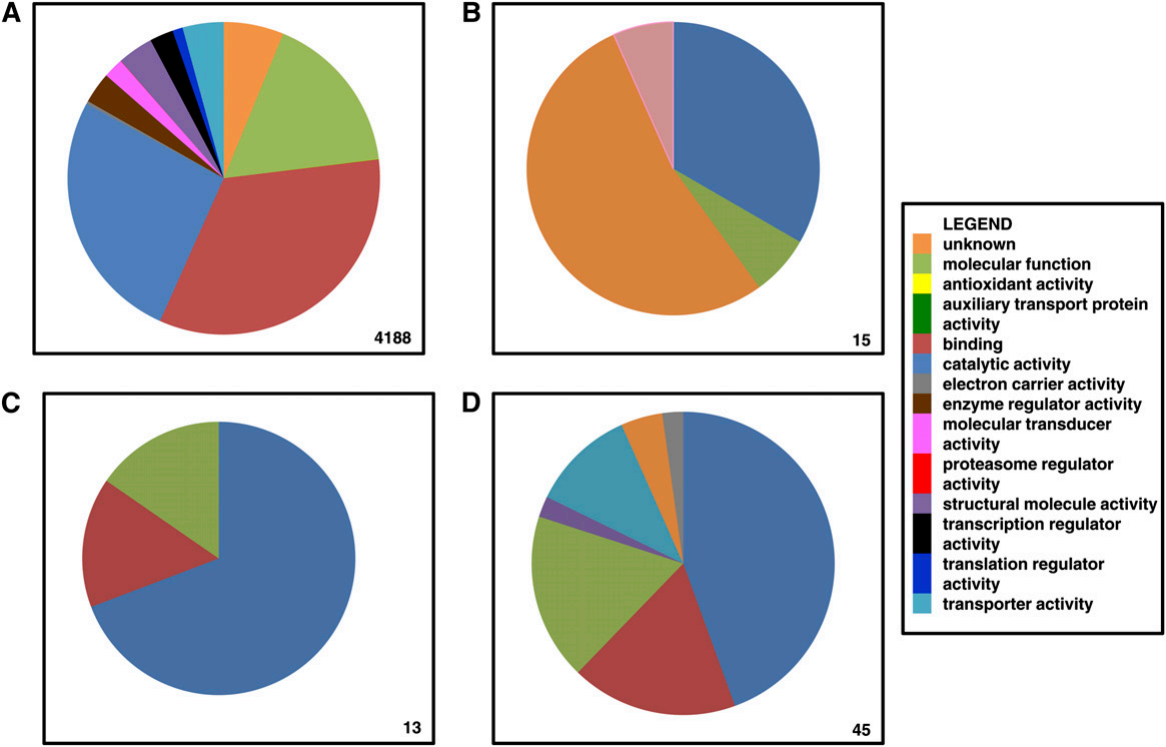
Comparison of function−parent attribution for transcripts for basal metabolism and transcripts highly differentially accumulated after a bloodmeal. Functional parent attribution of the transcripts (A) either not differentially accumulated or minimally accumulated (≤ 2-fold) after a bloodmeal and between sugar-fed mosquitoes of two strains, (B) significantly found only in B mosquitoes, (C) the top 1%, and (D) 5% of transcripts with the greatest-fold changes in accumulation between B and S mosquitoes in all three strains. The number of transcripts considered in each panel is on the bottom-right corner.

A total of 4188 transcripts exhibit no or small variation (≤2-fold) among S samples of the three strains and between B and S samples within each strain and correspond most likely to genes that encode products necessary for vital and/or basal metabolic functions (Table S2). Function−parent attributions for this group of transcripts revealed a predominance of those encoding proteins with binding activity over transcripts with molecular function and catalytic activity (Figure 1). Functional description is available for 2262 of these 4188 transcripts (Lawson *et al.* 2009) (Table S2). More than 10 transcripts were found associated with each of the following classes: protein recycling (ubiquitin and proteasome), zinc finger proteins, WD-repeat proteins associated with diverse functions (*i.e.* RNA-processing complexes, transcription, cytoskeleton assembly), ribosome proteins, signal transduction (GTP binding proteins, G coupled proteins), redox processes (cytochrome), translation (eukaryotic translation initiation factor), transcription (transcription factors, mediator complex), membrane trafficking (rab), cell growth, differentiation and survival (ras), and immunity (PIWIs, autophagy proteins, galectins, TOLL, IMD, and JACK-STAT pathway signaling members).

### Transcripts accumulated differentially among strains in sugar-fed mosquitoes

Approximately 25% of all analyzed transcripts accumulated differentially in S mosquitoes of different strains. However, in the majority of cases the differences observed were lower than 2-fold (Table 2 and Table S3). Detailed information on all pair-wise comparisons between S mosquitoes is presented in the accompanying supporting information. Here we describe only differences between the DEN-refractory Rex-D and the more permissive CTM strain. Totals of 2992 and 2080 transcripts accumulated more in Rex-D or CTM, respectively. Six were accumulated more than 5-fold in CTM with respect to Rex-D, including four of unknown function, one (AAEL005534-RA) annotated as a gonadotropin-inducible transcription factor, and one (AAEL014188-RA) annotated as a serine-type endopeptidase. In contrast, 310 transcripts accumulated ≥5-fold in Rex-D with respect to CTM. Functions could be attributed to 51% of these and they include aldehyde oxidases, cytochrome P450s, G-coupled receptors, *N*-acetylgalactosaminyltransferases, and nicotinic acetylcholine receptors. Immunity-associated transcripts accumulated differently in CTM and Rex-D S mosquitoes include five C-type lectins (AAEL008681-RA [CTL12], AAEL012353-RA [CTL15], AAEL005482-RA [CTL18], AAEL013566-RB [CTLGA2], AAEL017484-RA [CTLGA4]), two heme peroxidases (AAEL00376-RA [HPX4], AAEL002354-RA), one class B scavenger receptor (AAEL010655-RA [SCRBSP2]), a fibrinogen-related protein (AAEL008646-RA [FREP10]), and a Toll-like receptor (AAEL000671-RA [TOLL6]).

**Table 2:** Transcripts accumulated differentially between sugar-fed mosquitoes of two strains are classified on the basis of the fold-changes in accumulation (A). Some of these transcripts also were accumulated differentially after blood-feeding (B)

A. Comparison between S mosquitoes												
	Fold-Changes*^a^*											
	>10,000	>1000	>100	>10	10 − >5	5 − 2		<2	Total	Detected Only in One Strain		
Rex-D > CTM	0	0	3	131	176	791		1891	2992	Rex-D = 180		
CTM > Rex-D	0	0	0	0	6	191		1883	2080	CTM = 59		
									5072			
LVP > CTM	0	0	9	159	183	744		1009	2104	LVP = 179		
CTM > LVP	0	0	0	52	112	867		2324	3355	CTM = 58		
									5459			
LVP > Rex-D	0	1	0	39	69	364		680	1153	LVP = 27		
Rex-D > LVP	0	0	0	43	60	523		2235	2861	Rex-D = 48		
									4014			
B. Number of transcripts accumulated significantly 5hPBM among the ones accumulated differentially between S mosquitoes												
	Fold-changes*^a^*											
	>2							<2				
Rex-D > CTM	Rex-D = 186, CTM = 341, both = 231							Rex-D = 163, CTM = 591, both = 975				
CTM > Rex-D	Rex-D = 14, CTM= 101, both = 21							Rex-D = 175, CTM = 611, both = 795				
LVP > CTM	LVP = 251, CTM = 210, both = 331							LVP = 101, CTM = 170, both = 631				
CTM > LVP	LVP = 204, CTM = 2084, both = 367							LVP = 414, CTM = 292, both = 1491				
LVP > Rex-D	LVP = 155, Rex-D = 32, both = 174							LVP = 133, Rex-D = 45, both = 443				
Rex-D > LVP	LVP = 187, Rex-D = 67, both = 206							LVP = 832, Rex-D = 123, both = 1126				

CTM, Chetumal; LVP, Liverpool; Rex-D, Rexville D-Puerto Rico.

a Fold-changes between sugar-fed mosquitoes (A) and blood- and sugar-fed mosquitoes (B).

### Transcriptional responses to a bloodmeal

LVP, CTM, and Rex-D strains raised under identical conditions yielded S mosquitoes of similar weights (Table S4). CTM mosquitoes weighed significantly less than Rex-D upon imbibing blood and up to 1 hPBM. Totals of 6187, 7445, and 4649 transcripts accumulated differentially between B and S mosquitoes in the LVP, CTM, and Rex-D strains, respectively (Table S5). Quantitative reverse transcriptase, gene amplification, *i.e.*, polymerase chain reaction (qRT-PCR) was performed on a selection of 13 transcripts and RNA from CTM, Rex-D, and LVP mosquitoes. The qRT-PCR results validated the RNA-seq data, with only two exceptions (transcript AAEL011871-RA in CTM and AAEL008848-RA in Rex-D; Figure S2). To assess the similarity of the RNA-seq data with the qRT-PCR measurements, we calculated Pearson correlations of 0.97 (*P* < 0.001), 0.85 (*P* < 0.001), and 0.90 (*P* < 0.01) for LVP, CTM, and Rex-D, respectively. *T*-test values were 2.18 (*P* = 0.148), 2.18 (*P* = 0.453), and 2.18 (*P* = 0.188), respectively, for the three strains (Table S6).

**Figure 2:**
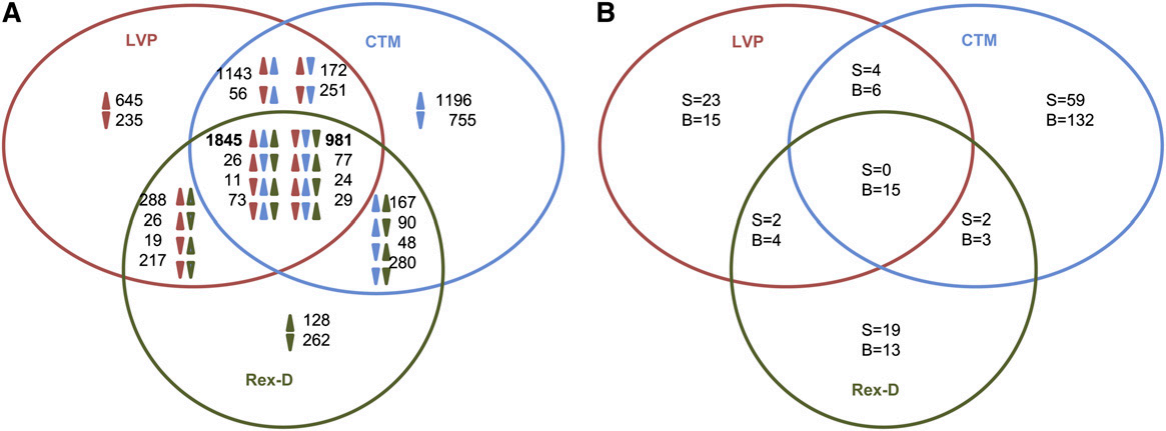
Venn diagram showing transcripts accumulated differentially between S and B mosquitoes 5 hPBM (A) and significantly found only in S or B mosquitoes (B) among the three *Ae. aegypti* strains analyzed.

The accumulation of 1845 and 981 transcripts consistently increased and decreased, respectively, in all three strains after a bloodmeal (Figure 2, Table S7). In general, Rex-D exhibited the lowest fold-changes and the smallest number of transcripts accumulated highly after the bloodmeal (Table 3). This observation supports the interpretation that CTM mosquitoes respond quicker or more intensively to a bloodmeal than LVP and Rex-D. To test this hypothesis, the expression profiles of five transcripts were analyzed by qRT-PCR at 5, 8, 12, and 24 hPBM. Results for four of the five transcripts tested showed a greater response of CTM to a bloodmeal compared with mosquitoes of the other two strains (Figure S3). Variability in the magnitude of changes in transcript accumulation after a bloodmeal was observed among the biological replicates when samples from the same strain were tested (Table S8). These results support the conclusion that the time between 5 and 8 hPBM is a dynamic metabolic period.

**Table 3:** Number of transcripts accumulated differentially between sugar- and blood-fed (5 hPBM) mosquitoes classified by fold-changes in accumulation level

** **	Fold							
Strain	>10,000	>1000	>100	>10	10 − >5	5 − 2	<2	Total
A								
LVP	0	5	9	42	59	959	771	1845
CTM	2	4	8	37	91	722	981	1845
Rex-D	0	1	9	46	68	281	1440	1845
B								
LVP	0	0	5	45	95	470	366	981
CTM	0	0	3	61	110	457	350	981
Rex-D	0	0	0	31	80	452	418	981
C								
LVP	0	0	0	4	16	218	407	645
CTM	0	0	2	81	116	518	479	1196
Rex-D	0	0	1	8	12	44	63	128
D								
LVP	0	0	0	25	36	113	61	235
CTM	0	0	0	58	78	357	262	755
Rex-D	0	0	0	17	28	106	111	262

LVP, Liverpool; CTM, Chetumal; Rex-D, Rexville D-Puerto Rico; PBM, post-bloodmeal.

A indicates 1845 transcripts with increased accumulation in all three strains; B, 981 transcripts with decreased accumulation in all three strains; C, transcripts with strain-specific increased accumulation; and D, transcripts with strain-specific decreased accumulation.

Fifteen transcripts were detected by RNA-seq only after a bloodmeal in all three strains (Figure 2). Four of these (AAEL013127-RB, AAEL009166-RA, AAEL013118-RA, and AAEL001621-RA) were confirmed to be present exclusively in B mosquitoes by reverse transcriptase gene amplification (RT-PCR; Table S9). RT-PCR also validated RNA-seq data on transcripts accumulated highly after a bloodmeal (Table S9, Figure S4). It is worth noting that many of the transcripts analyzed by RT-PCR also were detected in early developmental stages and in adult males. The absence of amplification products for transcripts AAEL009166-RA and AAEL001621-RA in CTM and Rex-D mosquitoes result from strain-specific sequence variation in the regions where primers were designed, as confirmed by the alignment of CTM and Rex-D reads to the reference LVP transcriptome (Figure S5).

**Figure 3:**
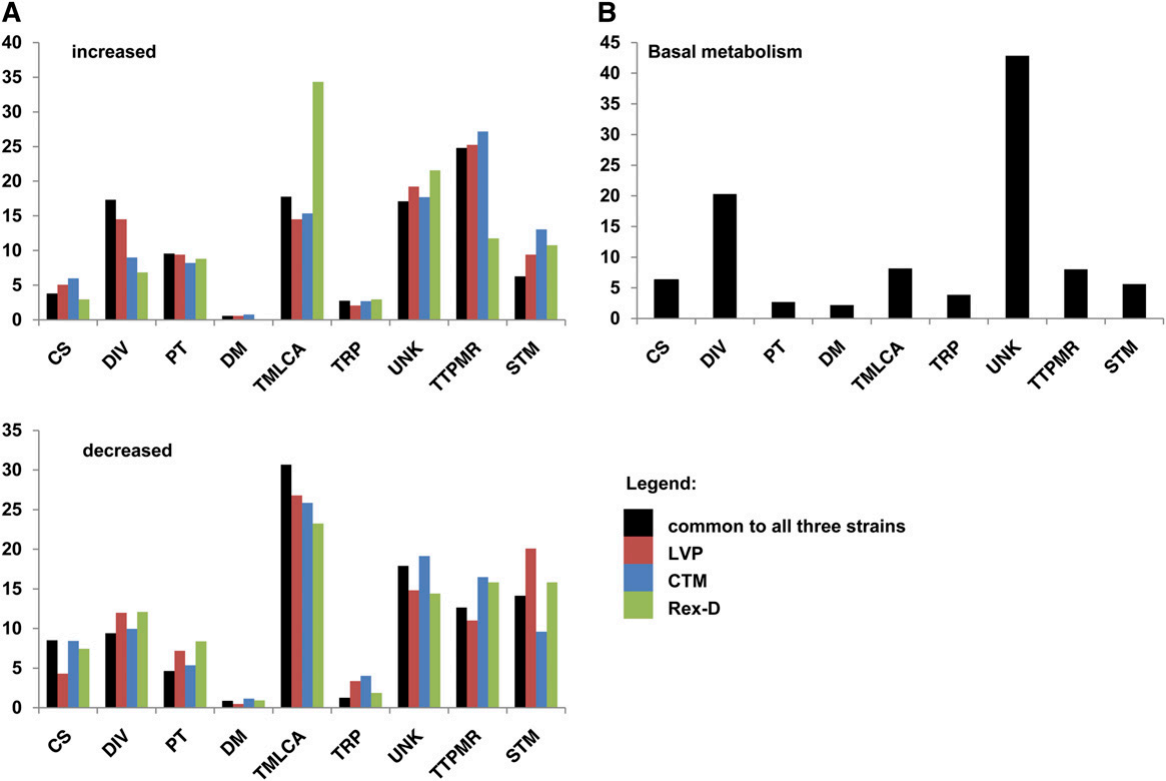
Percentages of functional group classifications for the transcripts accumulated differentially at 5 hPBM (A) and related to basal metabolism (B). Functional groups abbreviations are as follows: cytoskeleton and structural (CS), diverse function (DIV), protein turnover and chaperones (PT), defense mechanism (DM), transport and metabolism of lipids, carbohydrate and amino acids (TMLCA), transport and intracellular trafficking (TRP), unknown (UNK), transcription, translation, posttranslational modification and RNA processing (TTPMR), and signal transduction mechanism (STM). Data relative to transcripts found expressed only in B or S mosquitoes are included with those related to transcripts increased or decreased, respectively, following a blood meal.

The predicted functional classes with the largest representation among the proteins encoded by 1845 transcripts accumulated following a bloodmeal in all three strains were binding or catalytic activities (Figure S6). Transcripts within each of the most-represented functional classes identified were subclassified into functional groups. The majority of transcripts increased consistently in accumulation after a bloodmeal are attributed a role in transcription, translation, posttranscriptional modification or RNA processing, followed by metabolism of lipids, carbohydrate and amino acids (Figure 3). Most of these transcripts showed <5-fold differential accumulation between B and S mosquitoes.

A total of 981 transcripts decreased in accumulation after the bloodmeal in the three strains analyzed (Figure S6), with functional classes related to transport and metabolism of lipids, carbohydrates and amino acids, transcription, translation, posttranscriptional modification, and RNA processing and signal transduction represented most (Figure 3). The magnitudes of decreased accumulation between S and B mosquitoes were smaller than those observed for increases, with only eight transcripts (5 in LVP, 3 in CTM) showing >100-fold decreases after a bloodmeal (Table 3).

Despite the observation that the same functional classes are represented within groups of transcripts that increased and decreased in accumulation after a bloodmeal, the numbers of transcripts associated with the various functions differed significantly (Table S10). The transcripts accumulated after a bloodmeal were enriched in functions related to protein-turnover and chaperones, transcription, translation, posttranslational modification, and RNA processing. These transcripts encode proteins associated with catalytic activities, in transport and metabolism of lipids, carbohydrates and amino acids, and related to blood digestion and the progression of the gonotropic cycle (supporting information). Many of the transcripts showing the greatest decrease in accumulation after a bloodmeal in all three strains encode structural proteins.

The number of strain-specific transcripts varying highly (<10-fold) in abundance between B and S mosquitoes is smaller in general than those found in all three strains (Table 3). CTM mosquitoes show both the greatest number of strain-specific, differentially accumulated transcripts (Figure 2) and the largest range of changes in transcript accumulation levels. Overall, the largest proportion of transcripts showing strain-specific increased accumulation after bloodmeal are linked to binding and catalytic functions and associated with transcription, translation, and posttranslational modification, whereas the largest proportion of strain-specific decreased transcripts are linked to transcription, translation, posttranscriptional modification, and RNA processing in LVP and CTM and to transport and metabolism of lipids, carbohydrates, and amino acids in Rex-D (Figure S6).

**Figure 4:**
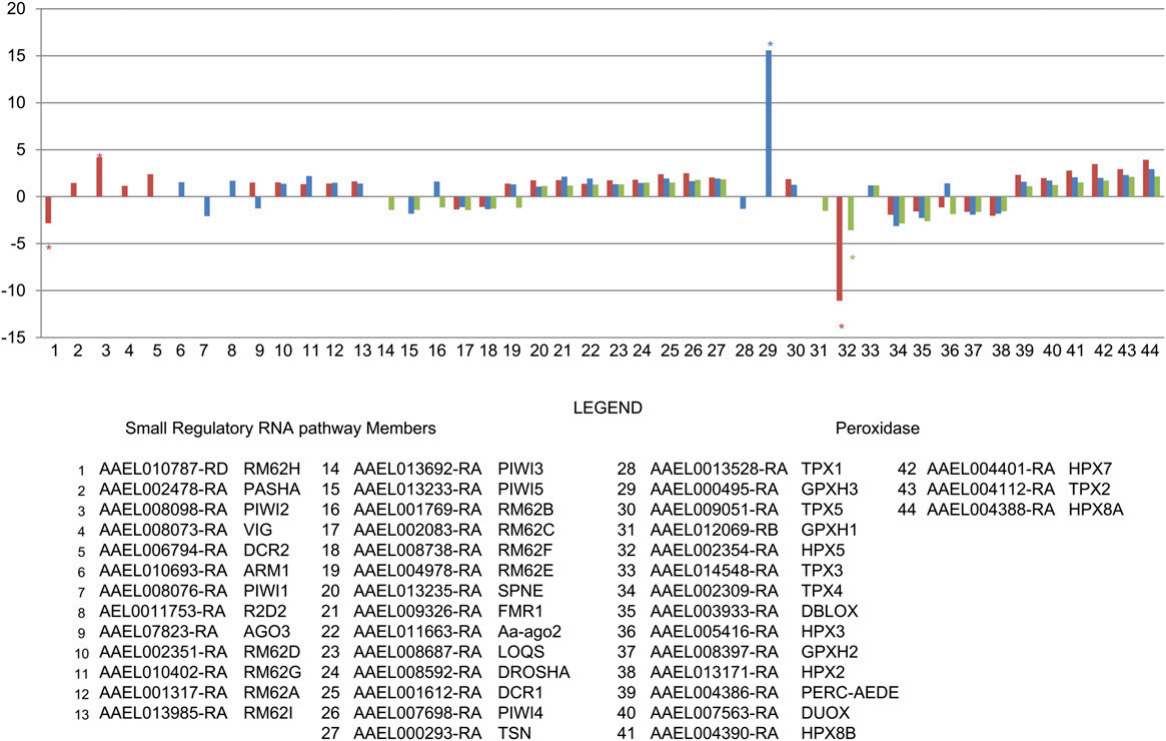
Immunity-related transcripts. The significant fold-changes in accumulation 5 hPBM between B and S mosquitoes for SRRP members (1−27) and transcripts associated with peroxidase activity (28−44) are shown in the *Ae. aegypti* strain LVP (red), CTM (blue), and Rex-D (green). The asterisk indicates transcripts with ≤100 RNA-seq reads mapping in both the S and B sample.

Although detailed information on all transcript functions and accumulation levels in each strain is presented in the accompanying supplemental information (supporting information and Table S6), information on transcripts associated with immunity is elaborated here. Totals of 168, 178, and 223 of the 471 immunity-associated transcripts are accumulated differentially between S and B mosquitoes in Rex-D, LVP, and CTM, respectively (Table S11). The majority of the immunity-associated transcripts decreased in accumulation after bloodmeal. CTM showed the greatest number of these transcripts accumulating differentially after a bloodmeal (97 increased, 119 decreased, 6 expressed only in B, and 1 only in S mosquitoes) and also showed the widest ranges in change of transcript accumulation after a bloodmeal (+33.5-fold for AAEL000625-RA [Cecropin F, CecF] to −39-fold for AAEL003389-RA [attacin, AaATT]). Differences in the classes of immunity-associated transcripts accumulated differentially after a bloodmeal among strains were observed. In addition, the magnitude and direction of change in accumulation for a specific transcript may differ among strains after a bloodmeal (Figure 4, Table S12). For example, transcripts AAEL00495-RA (peroxidase GPXH3) accumulate as much as 15.6-fold after a bloodmeal in CTM and AAEL002354-RA (HPX5) decreases 11.1-fold in LVP, but both only change modestly or are not accumulated at different levels between S and B in the other strains analyzed.

The members of the small interfering RNA (siRNA) pathway, Dicer-2 (AAEL006794-RA [DCR2]), Argonaut 2 (AAEL011663-RA [Aa-ago2]), and R2D2 (AAEL011753-RA), are proposed to comprise the main antiviral response in mosquitoes (Brackney *et al.* 2010; Campbell *et al.* 2008; Cirimotich *et al.* 2009; Myles *et al.* 2009; Sanchez-Vargas *et al.* 2009; Scott *et al.* 2010). Only transcripts of the Aa-ago2−encoded endonuclease accumulated significantly in all three strains after a bloodmeal, but <2-fold (1.4, 1.9, and 1.3-fold in LVP, CTM, and Rex-D, respectively). Dicer2 accumulated significantly in LVP (2.4-fold) but was not differentially accumulated in either CTM or Rex-D. R2D2 increased significantly in accumulation in CTM (1.7-fold) but was not differentially accumulated in LVP and Rex-D.

The microRNA (miRNA) pathway regulates gene expression by mRNA cleavage or transcriptional repression (Mukherjee and Hanley 2010). Three members of this pathway, AAEL001612-RA (Dicer-1), AAEL008592-RA (Drosha), and AAEL008687-RA (Loquacious), accumulate significantly in all of the strains after a bloodmeal. Dicer-1 accumulates 2.4-, 1.9-, and 1.5-fold; Drosha 1.8-, 1.5-, and 1.5-fold; and Loquacious 1.8-, 1.3-, and 1.3-fold in LVP, CTM, and Rex-D, respectively (Table S11).

Three classes of scavenger receptors, A, B, and C, differ in binding-domain properties (Waterhouse *et al.* 2007). Three (AAEL014367-RA [SCARSP3], AAEL009192-RA [SCRASP1], AAEL001941-RA [SCRAC1]) of four class A scavenger receptor members accumulated significantly after a bloodmeal in CTM but decreased or did not change significantly in abundance in LVP and Rex-D. The 14 members of class B scavenger receptors generally show decreases in post-bloodmeal accumulation. This is evident particularly in CTM mosquitoes, where 6 of 14 transcripts decreased significantly >2-fold. Two members of class C scavenger receptors, AAEL006355-RA (SCRC1) and AAEL006361-RA (SCRC2), which are specific to insects and are proposed to function as pattern recognition receptors (PRRs) in phagocytosis and innate immunity (Waterhouse *et al.* 2007), decreased ≤4.3-fold after a bloodmeal in all three strains.

Transcripts encoding caspases tended to decrease in accumulation in all three strains after a bloodmeal. CTM had five (AAEL005963-RA [CAPS15], AAEL003444-RA [CASPS19], AAEL003439-RA [CASPS18], AAEL005956-RA [CASPS16], and AAEL017498-RA [CASPS21]) that showed significant decreases (1.4- to 5.3-fold). In contrast, inhibitors of apoptosis (IAPs) encoding transcripts increased slightly (1.2- to 3.2-fold) in all three strains, except AAEL009074-RA (IAP1), which decreased in all three strains (−1.9, −2.5, and −2.1 in LVP, CTM, and Rex-D, respectively) and AAEL012512-RA (IAP9), which decreased (−5.1-fold) in LVP.

### Protein network analysis of bloodmeal-induced changes in transcript accumulation

By using a Markov Cluster algorithm, Guo *et al.* (2010) developed a protein interaction network in which 3500 *Ae. aegypti* proteins are organized into 494 functional modules. A total of 2465 of these proteins are encoded by transcripts exhibiting significant differential accumulation between B and S mosquitoes in at least one of the three strains analyzed (Figure S7, Table S13). Modules enriched in Gene Ontology terms for cytoplasm organization and biogenesis, ribosome biogenesis and assembly, transcription, DNA metabolic processes, response to endogenous stimuli, and cell cycle were enriched significantly with proteins encoded by transcripts increased in accumulation in all three strains after a bloodmeal (Table S14). A module enriched in Gene Ontology terms for cytoskeleton organization and biogenesis, reproduction, and cell differentiation was enriched significantly with proteins corresponding to transcripts consistently decreased in accumulation following a bloodmeal. Results from the protein network analyses are consistent with those from functional attribution of the differentially accumulated transcripts.

### Metabolic pathways involving transcripts differentially accumulated post-bloodmeal

**Figure 5:**
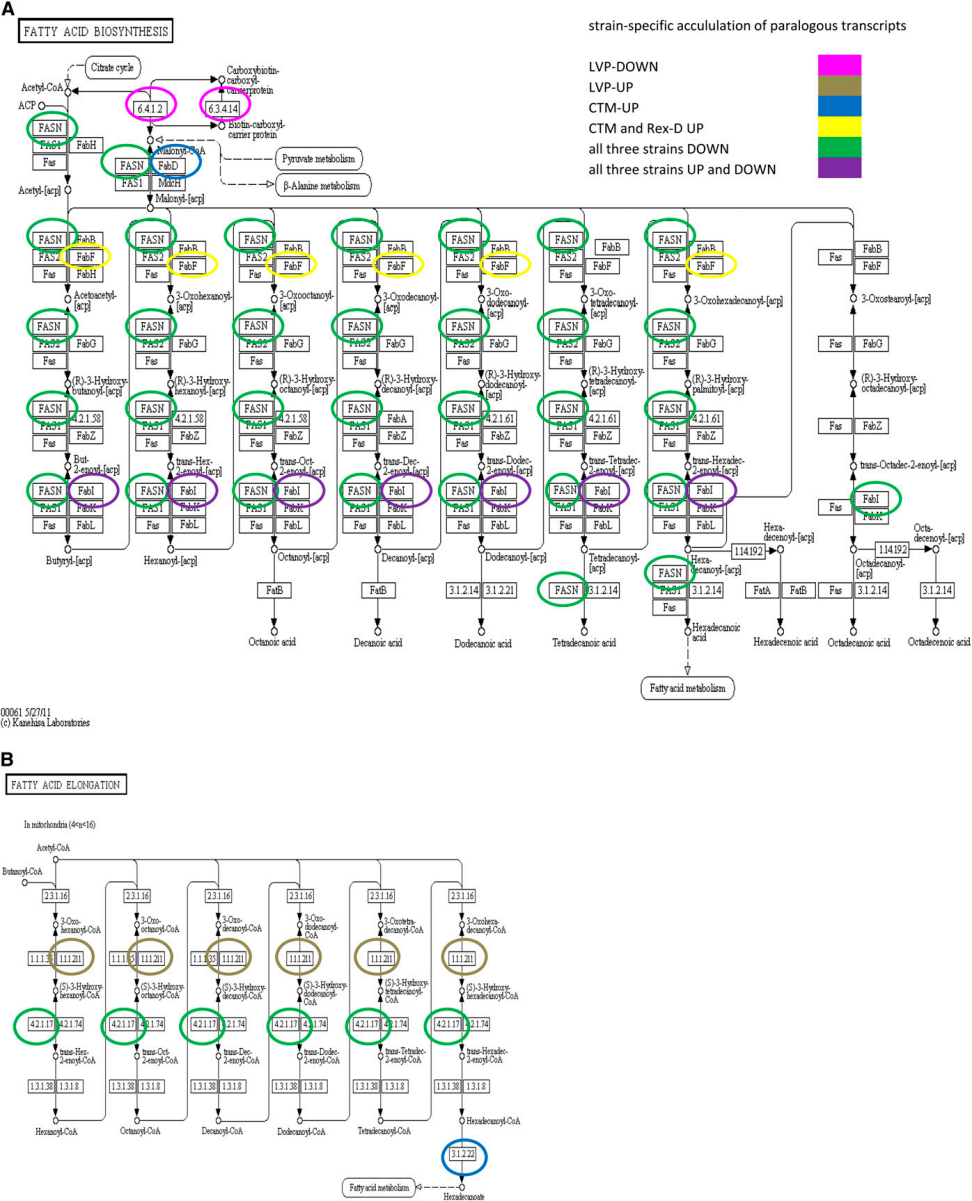
Fatty acid biosynthesis and elongation in mitochondria. Proteins corresponding to transcripts accumulated in a strain-specific manner are circled in a color corresponding to the strain and condition defined in the legend (panels A and B).

Metabolic pathways correlated with transcripts increased in accumulation after a bloodmeal in all three strains were visualized in LinkinPath (Ingsriswang *et al.* 2011) alongside pathways associated with transcripts accumulated in a strain-specific manner and transcripts decreased in accumulation after a bloodmeal (Figure S8 and Figure S9). As seen with the function−parent attribution analyses, the transcripts increased and decreased in accumulation are associated with proteins involved in similar pathways. However, differential expression of paralogous genes among the strains was observed that could account for the intensity and/or the rate of pathway activation/deactivation after a bloodmeal. For example, biosynthesis of fatty acids associates predominantly with transcripts decreased in accumulation in all three strains after a bloodmeal, but the elongation of fatty acids in mitochondria was exclusively associated with transcripts increased in accumulation (Figure 5). Proteins corresponding to transcripts accumulated differentially in a strain-specific manner were identified in both cases, with the same protein function encoded by paralogous transcripts in the different strains. Examples include FabI (enoyl-[acyl carrier protein] reductase) of the fatty acid biosynthesis pathway linked to transcripts AAEL003961-RA and AAEL002493-RA, which significantly increased in all three strains (fold-changes: 1.4-2.4); AAEL009634-RC and AAEL014840-RA, both of which increased 2-fold in CTM; AAEL000705-RA, AAEL000689-RA and AAEL004273-RA, which decreased in CTM (fold-changes: 2.5-4.4); AAEL009685-RB, which decreased 5.4-fold in Rex-D; and AAEL017302-RA, AAEL007669-RA, AAEL008227-RA, AAEL009625-RA, AAEL013491-RA, AAEL017320-RA, AAEL000690-RA, AAEL002228-RA, AAEL003148-RA, AAEL003183-RA, AAEL010075-RA, AAEL012400-RA, and AAEL008016-RA, which decreased in all three strains, with fold-changes in the range of 0.6-11.3. All of these transcripts are annotated currently as short-chain and steroid dehydrogenases, oxidoreductases, or fatty acid synthases (AegyXcel) and match to short-chain dehydrogenases in the PFAM database (Finn *et al.* 2008), with the exception of AAEL002228-RA, whose best match is ketoacyl synthase. They all have the PKS_KR enzymatic domain as a best match in the SMART database (Letunic *et al.* 2009), except for AAEL012400-RA, which matches the SEP enzymatic domain. The protein HADHA (4.2.1.17 in the fatty acid elongation pathway), functioning as an aldehyde reductase and enoyl-coA hydratase, is associated with transcripts AAEL010146-RB (annotated as 3-hydroxyacyl-coa dehydrogenase) and increased 2.9-fold in LVP and AAEL003993-RA (putative cyclohex-1-ene-1-carboxyl-CoA hydratase), which increased in all three strains with fold-changes ranging between 1.6 and 2.3.

Another example of differential accumulation of transcripts representing paralogous genes is seen in the lipase gene family. Three genes (AAEL001837, AAEL007055, AAEL14551) of the 72 annotated currently as lipases correspond to transcripts accumulated differentially after a bloodmeal in all three strains (fold-changes: −7.5 to 4.3) and eight to transcripts accumulated differentially in a strain-specific manner. Two (AAEL002909-RA, AAEL001076-RA) of these latter transcripts were found exclusively in S and one (AAEL006970-RA) only in B CTM mosquitoes. The remaining had fold-changes ranging from −2.2 to 2.6. No reads were mapped in any sample for 21 lipase genes, which may indicate that they are expressed exclusively in adult males or during pre-adult stages of *Ae. aegypti* development. Alternatively, these predicted genes could be pseudogenes.

Chitin has been associated with mosquito responses to a bloodmeal, including the digestive processes (Benoit *et al.* 2011; Billingsley and Rudin 1992). Chitin and proteins are the main components of the peritrophic matrix (PM) (Lehane 1997). The PM is newly synthesized after each bloodmeal and becomes evident 4-8 hPBM in *Ae. aegypti* (Perrone and Spielman 1988). PM increases digestion efficiency and provides a physical barrier that prevents or reduces pathogen invasion (Kato *et al.* 2008). Two genes (AAEL005618 and AAEL002718) encoding chitin synthases are in the current annotation of the *Ae. aegypti* genome. Hybridization *in situ* studies were interpreted to show an increased accumulation of AAEL005618-RA in the midgut epithelial cells after a bloodmeal (Ibrahim *et al.* 2000). In our work, transcript AAEL005618-RA was decreased in accumulation after a bloodmeal in all three strains (-3.2 in LVP; −2.9 in CTM; −2.4 in Rex-D). Transcript AAEL002718-RA was not accumulated differentially in Rex-D but decreased in accumulation in LVP (0.22-fold) and increased in CTM (4.14-fold). The expression of these transcripts in the midgut of blood fed mosquitoes need to be determined to resolve these results.

Heat-shock proteins (HSPs) act as molecular chaperones and help preserve the function of other proteins (Feder and Hofmann 1999). An increased accumulation of heat shock protein 70 (HSP70) was detected as early as 1 hPBM and its presence is attributed to protection from the stress of warm blood ingestion (Benoit *et al.* 2011). HSPs are encoded by a gene family that has expanded in the *Ae. aegypti* genome (Waterhouse *et al.* 2008). Six *Ae. aegypti* Hsp70 genes were identified (Gross *et al.* 2009), only two of which, AaHsc70-4 (AAEL016995-RA) and AAHsc-3a/b (AAEL017349-RA), correspond to annotated transcripts. AAEL016995-RA decreased (1.4- to 1.7-fold) and AAEL017349-RA increased (2.3- to 3.2-fold) in accumulation in all three strains. RNA-seq reads also mapped to the other characterized hsp70 encoding genes (Gross *et al.* 2009). Four of these (AaHsc70-2, AaHsc70-4, AaHsc-3b, and AaHsc-3a) showed a number of reads *per* strain from 35 to 17,233, with a greater number of reads in the B mosquitoes. Three genes (AaHsp70Bb, AaHsp70Bb’ and AaHsp70Ca’) had no reads mapping in any sample, whereas <10 reads mapped to the remaining genes.

## Discussion

The results reported here reveal that the transcriptomes of *Ae. aegypti* mosquitoes from distinct strains vary significantly in complexity and abundance of specific transcripts. This variation is evident in nonblood-fed mosquitoes and is enhanced after a bloodmeal. CTM showed a larger number of differentially abundant transcripts after a bloodmeal and a wider range of fold-changes than Rex-D and LVP. Transcriptional differences among strains appear unrelated to initial mosquito size because S mosquitoes did not differ in weight among strains. In contrast, CTM mosquitoes did weigh less than Rex-D just after a blood meal. Mosquito weight after a bloodmeal is affected by diuresis (Stobbart 1977). Excretion of water and ions starts during blood-feeding (Williams *et al.* 1983) and is thought to be under the control of an uncharacterized neuropeptide hormone produced in the head and acting on the Malpighian tubules (Wheelock *et al.*1988). Because CTM mosquitoes showed an overall more intense transcriptional response to a bloodmeal than LVP and Rex-D, we cannot exclude the possibility that the difference in weight detected after blood-feeding is due to a quicker diuresis in CTM. Three transport channel proteins, aquaporins (AaAQP) 1, 4, and 5, have been linked to diuresis (*Ae. aegypti* has six annotated AaAQP genes) (Drake *et al.* 2010). AaAQP1 and 4 were decreased significantly in accumulation in all three strains after a bloodmeal (fold changes: 4.1-34.3), whereas the gene encoding AaAQP5 (AAEL005008) shows three splice variants, all of which were not differentially accumulated after feeding. The whole-body decrease in accumulation of AaAQP1 and 4 shown by RNA-seq after a bloodmeal is consistent with microarray results at 3 hPBM (Dissanayake *et al.,* 2010) but masks tissue-specific differential expression, primarily at the Malpighian tubules (Drake *et al.* 2010). The significant decrease in body weight observed in CTM blood-fed mosquitoes with respect to Rex-D also is compatible with the engorgement of a smaller bloodmeal by CTM. Hence, the more intense response to bloodmeal observed in CTM could be related to the need of optimizing the use of the resources provided by a smaller meal.

Transcripts associated with metabolism and transport of lipids, carbohydrate and amino acids, and the progression of the gonotropic cycles were among the most increased in accumulation after bloodmeal. Transcripts decreased most in accumulation after a bloodmeal are associated with structural components. Transcripts attributed functions in transcription, translation, and posttranslational protein modification also were highly differentially-accumulated, but many of these show strain-specific regulation. These observations support the hypothesis that important differences among the strains are conferred by distinct patterns of gene expression, protein synthesis and modifications. Strain differences also may result from selective expression of paralogous transcripts.

It is reasonable to hypothesize that the pattern of post-bloodfeeding transcriptome change characteristic of a specific strain could influence vector competence. This is supported by evidence that shows that permissiveness or barriers to DENV infection often occur early in infection (Black *et al.* 2002). CTM has the most pronounced changes after bloodmeal, is the most recently laboratory-adapted strain, and is the one with the greatest susceptibility to DENV2 infection (Salazar *et al.* 2007). Laboratory adaptation affects the level of polymorphism within a population because of the Wahlund effect (Wahlund 1928). We cannot exclude the possibility that the less-pronounced response of Rex-D to a bloodmeal is biased by the fact that it is an inbred strain that has been maintained in the laboratory for over 20 years (Miller and Mitchell 1991). However, LVP, which was the earliest field-derived strain among the three tested (Macdonald 1963), showed an intermediate response (both in terms of number of transcripts and fold-changes in accumulation) between CTM and Rex-D. Because the three mosquito strains used in this study were reared under identical laboratory conditions, differences detected are characteristic of each strain and reflect their unique geographic origins and colonization histories. It will be important to compare bloodmeal-induced changes in gene expression of field-derived *Ae. aegypti* mosquitoes from various geographic sites, including the Yucatán Peninsula and Puerto Rico, from where CTM and Rex-D originated, respectively, and correlate the data with susceptibility to DENV.

A close association between blood digestion and DENV susceptibility can be proposed on the basis of several observations. First, the timing of the events: DENV is ingested through a bloodmeal and within minutes invades midgut epithelial cells to replicate and mature before disseminating through the hemocoel to secondary tissues (Mosso *et al.* 2008; Salazar *et al.* 2007). DENV infection can be blocked at the time of midgut infection (midgut infection barrier) or dissemination (midgut escape barrier) (Black *et al.* 2002). Quantitative trait loci analyses of the midgut infection barrier identified three loci with additive effects that encompass several digestive enzymes including early and late trypsins, maltase, aminopeptidase N and carboxypeptidase A (Black *et al.* 2002; Gomez-Machorro *et al.* 2004). It was proposed that trypsin inhibition affects DENV2 replication in the midgut and virus dissemination (Molina-Cruz *et al.* 2005). Early trypsin may contribute to proteolytic processing of DENV2 surface proteins (Gorrochotegui-Escalante *et al.* 2005). However, no association has been discovered between quantitative trait nucleotides in the early trypsin gene *try3_aedae* (AAEL007818) and susceptibility to DENV2 in *Ae. aegypti* populations from Mexico (Gorrochotegui-Escalante *et al.* 2005). RNA-seq data confirms a decrease in accumulation for transcript AAEL007818-RB at 5 hPBM, a splice variant of the early trypsin gene (Noriega and Wells 1999). The fold-decrease in accumulation spans one order of magnitude, from −23.55 in Rex-D to −130.24 in CTM and −138.24 in LVP, supporting the hypothesis that differences in early trypsin regulation may be associated with distinct DENV2 susceptibility across *Ae. aegypti* strains.

Digestion and immunity share bio-products such as reactive oxygen species (ROS) (Molina-Cruz *et al.* 2008), and these processes are linked at the protein network level (Guo *et al.* 2010). The majority of immunity-related genes were decreased in accumulation in all three strains after a bloodmeal. The most pronounced decrease in accumulation was observed for the antimicrobial peptide (AMP) attacin (AAEL003389-RA). Indeed, all AMPs decreased in accumulation with the exception of CecF (AAEL000625-RA), which significantly increased in accumulation exclusively in CTM. The overall AMP decrease may be associated with an increase in bacterial proliferation observed after a bloodmeal (Oliveira *et al.* 2011). The most common midgut bacteria do not show proteolysis activity but are implicated in the lysis of red blood cells, which release hemoglobin (Gaio *et al.* 2011). At the same time, hemoglobin negatively affects ROS production in the midgut, which triggers bacteria proliferation (Oliveira *et al.* 2011). Although ROS reduction potentially favors DEN infection (Oliveira *et al.* 2011), bacteria proliferation antagonizes it (Xi *et al.* 2008). Antioxidant activity by peroxidase and superoxide-dismutase varied across strains. CTM showed the greatest number of transcripts associated with antioxidant activity accumulated after a bloodmeal among the strains analyzed. In addition, S CTM mosquitoes accumulate greater levels of transcripts associated with antioxidant activity than Rex-D. CTM also had the largest number of P450 and glutathione-s transferase transcripts accumulated differentially after a bloodmeal, with increases in accumulation up to 15-fold (AAEL000325-RA). These results support a model of more intense antioxidant activity in B CTM than in Rex-D, which may facilitate DENV infection in the former.

Transcripts encoding proteins associated with autophagy, IAPs, IMD pathway, and SRRP members represent immunity genes that showed an exception to the overall decline in corresponding-transcript accumulation after a bloodmeal. Increases were overall within 2-fold. A notable exception is the IMD pathway member Caspar1 (AAEL014734-RA) that increased 4.7- to 5.8-fold in all strains. Caspar is a negative regulator of the IMD pathway (Kim *et al.* 2006); therefore, its up-regulation is consistent with the observed decrease in accumulation of AMPs.

In summary, the *Ae. aegypti* strains analyzed demonstrated variability in their transcriptomes before and after bloodmeal. Profiles differ in the number of transcripts detected, their level of accumulation in S mosquitoes, and in the changes in accumulation following the ingestion of a bloodmeal. Although these differences may result from distinct RNA turnover rates among strains, it is most likely a result of differential gene regulation. These data indicate the need for caution in making generalizations about individual gene expression profiles across different strains of *Ae. aegypti*. For example, constructs used in genetics-based strategies of vector control would require the previous analyses of cross-strain promoter activity (James *et al.* 2011). The differences observed encompass several transcripts associated previously with vectorial capacity to DENV. Future studies will investigate the transcriptomes of CTM and Rex-D mosquitoes infected with DENV2. Also, it will be necessary to assess the susceptibility of CTM and Rex-D to other DENV serotypes to determine whether or how their distinct transcriptional responses to bloodmeals described herein influence vector competence.

## Supplementary Material

Supporting Information
