# Supplementary material for: Strain Variation in the Transcriptome of the Dengue Fever Vector, Aedes aegypti
Source: G3 (Bethesda). 2012 Jan 1;2(1):103–14. doi: 10.1534/g3.111.001107 (PMC3276191; doi:10.1534/g3.111.001107)
Supplement: Supporting Information [file supp_2_1_103__index.html]

Supporting Information 

# Strain Variation in the Transcriptome of the Dengue Fever Vector, *Aedes aegypti*

## Supporting Information for Bonizzoni *et al.*, 2011

**Files in this Data Supplement:**

- Supporting Information - Figures S1-S9 and Tables S1-S14 (PDF, 2.3 MB)
- Figure S1 - Pearson correlation comparisons for RNA-seq replicates (PDF, 392 KB)
- Figure S2 - RNA-seq data validation by qRT-PCR (PDF, 84 KB)
- Figure S3 - Expression profile of five transcripts between 5 and 24 hours PBM in three Aedes aegypti strains (PDF, 80 KB)
- Figure S4 - Results of RT-PCR on 8 developmental stages of three strains of Ae. aegypti mosquitoes for transcripts (A) AAEL013584-RA and (B) AAEL10196-RA, (C) AAEL013712-RA , (D) AAEL013713-RA showing the PCR bands classification as N/A, +/-, + or ++ (PDF, 284 KB)
- Figure S5 - RNA-seq reads coverage (A) and RT-PCR primers position (B,C) in the three Ae. aegytpi strain tested for transcript AAEL009166-RA and AAEL001621-RA (PDF, 492 KB)
- Figure S6 - Function parent of differentially-accumulated transcripts (PDF, 92 KB)
- Figure S7 - Protein network. Aedes aegypti protein network (A) and derived functional modules (B) (Guo et al., 2010) (PDF, 320 KB)
- Figure S8 - Metabolic pathways corresponding to transcripts increased in accumulation 5hPBM in all three strains (allup), in LVP (Lup), in CTM (cup) and in Rex-D (Rup) are visualized by LinkinPath (Ingsriswang et al., 2011) (PDF, 444 KB)
- Figure S9 - Metabolic pathways corresponding to transcripts decreased in accumulation 5hPBM in all three strains (down\_all\_three), in LVP (lvp\_down), in CTM (ctm\_down) and in Rex-D (rexd\_down) are visualized by LinkinPath (Ingsriswang et al., 2011) (PDF, 88 KB)
- Table S1 - RT-PCR primers and conditions (PDF, 64 KB)
- Table S6 - Average fold changes of a random selection of thirteen genes in sugar- (S) and blood-fed (B) Ae. aegypti mosquitoes of three different strains as detected by qPCR (PDF, 80 KB)
- Table S8 - Fold-changes between blood- and sugar-fed Ae. aegypti mosquitoes of three different strains as detected by qPCR at 5, 8, 12 and 24 hPBM (PDF, 92 KB)
- Table S9 - Expression profile of eighteen Ae. aegypti transcripts during development (PDF, 88 KB)
- Table S10 - Comparison of number of transcripts from different functional classes (PDF, 84 KB)
- Table S12 - Enrichment of immunity-related classes of transcripts (PDF, 60 KB)
- Table S2 - List of transcripts accumulated differentially less than 2- fold and not accumulated differentially between sugar-fed mosquitoes across strains and between sugar- and blood-fed mosquitoes within each strain (.xlsx 3.2 MB)
- Table S3 - Differential accumulation of transcripts among sugar-fed mosquitoes (.xlsx 640 KB)
- Table S4 - Mosquito weight and blood-meal (.xlsx 20 KB)
- Table S5 - Actual Number of reads and normalized log2 fold-changes in accumulation of transcripts between blood- and sugar-fed mosquitoes of the Aedes aegypti LVP, CTM and Rex-D strains
- Table S7 - Strain-signature (.xlsx 80 KB)
- Table S11 - Immunity associated-transcripts (.xlsx 96 KB)
- Table S13 - Proteins in clusters from the high-confidence protein interaction network corresponding to the transcripts identified as accumulated differentially between B and S mosquitoes by RNA-seq data in at least one of the three Ae. aegypti strains analyzed (.xlsx 64 KB)
- Table S14 - Protein cluster enrichments (.xls, 148 KB)
