# Supplementary material for: Strain Variation in the Transcriptome of the Dengue Fever Vector, Aedes aegypti
Source: G3 (Bethesda). 2012 Jan 1;2(1):103–14. doi: 10.1534/g3.111.001107 (PMC3276191; doi:10.1534/g3.111.001107)
Supplement: Supporting Information [file supp_2.1.103_001107SI.pdf]

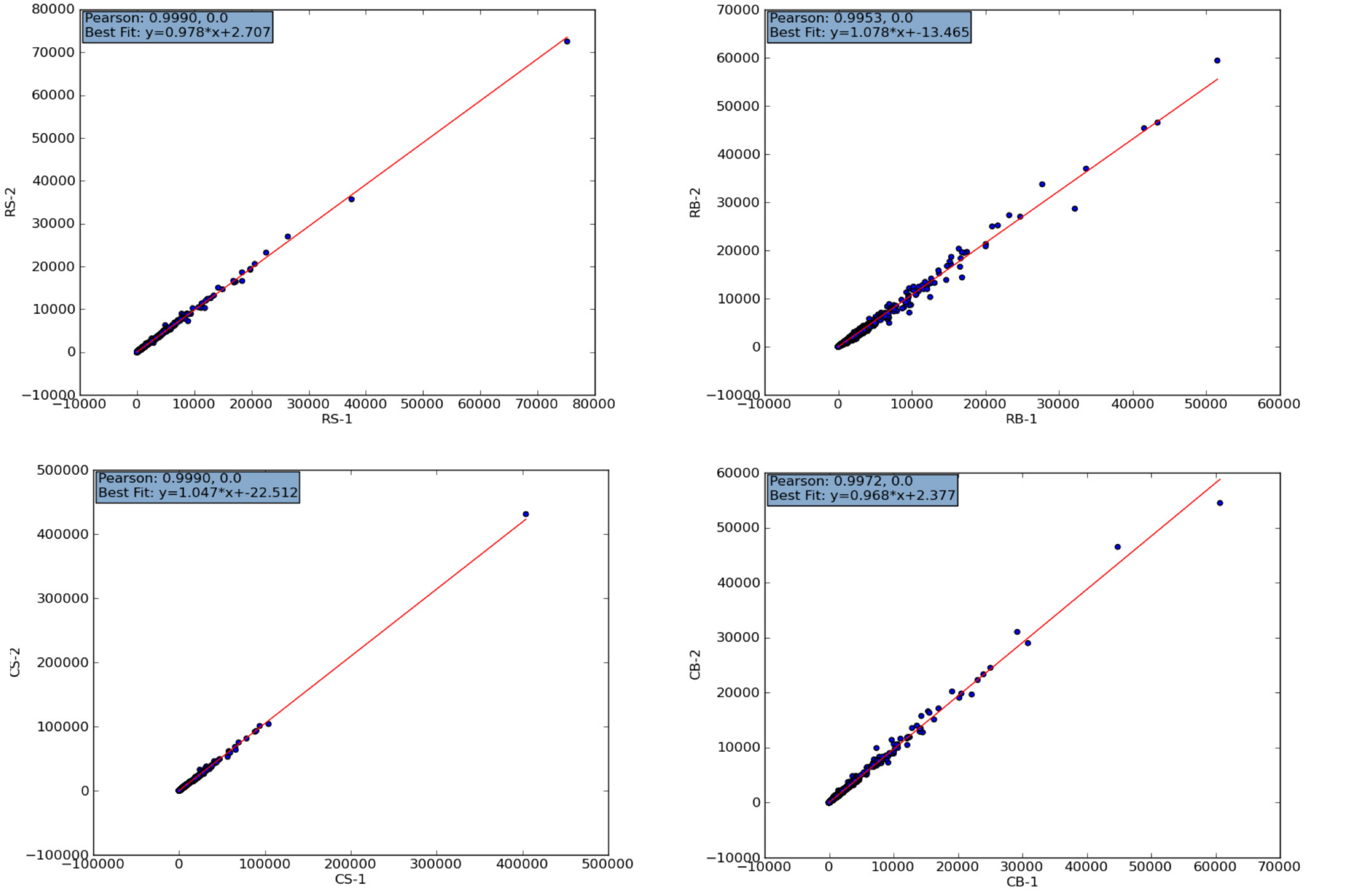

Figure S1 Pearson correlation comparisons for RNA-seq replicates.

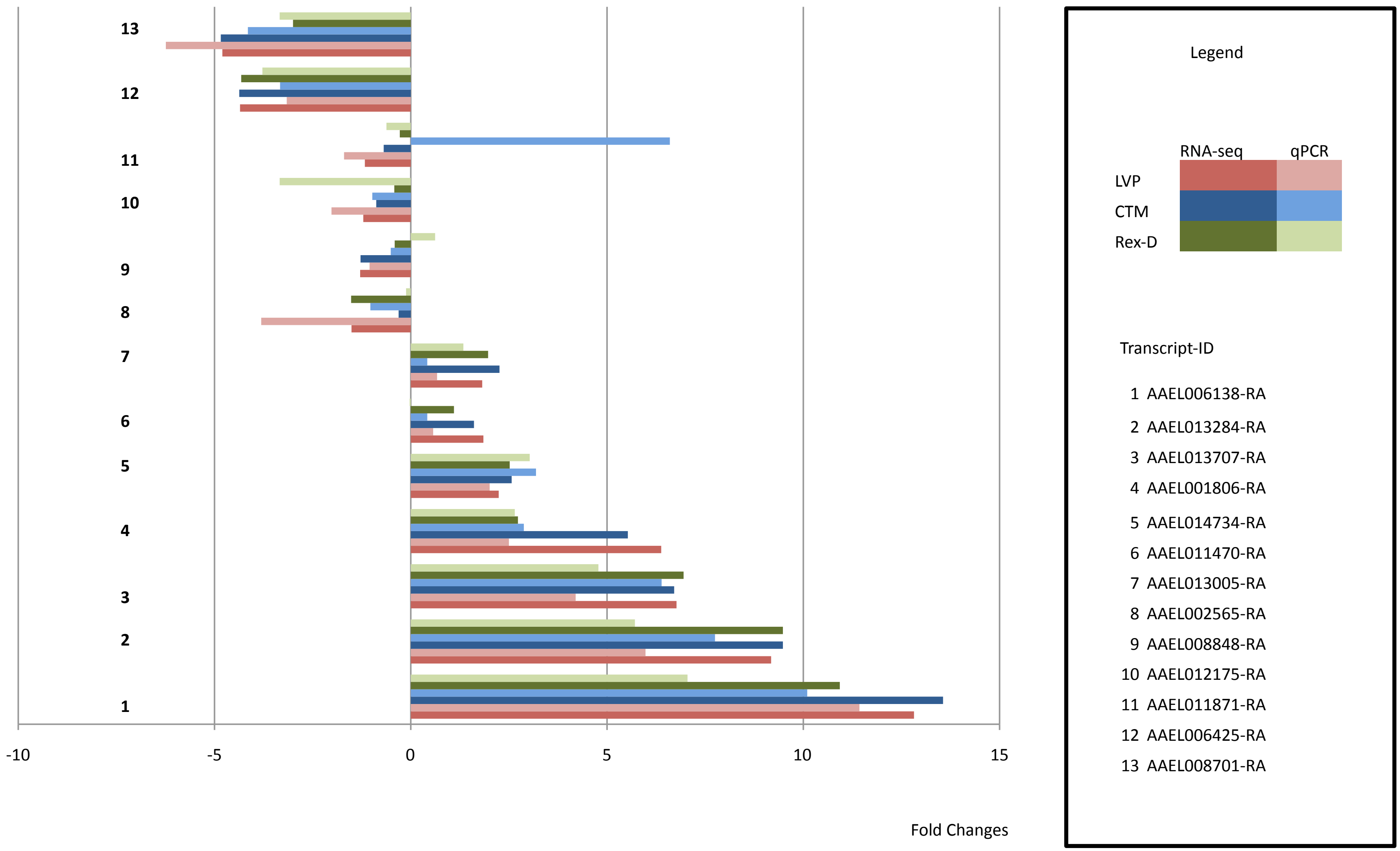

Figure S2 RNA-seq data validation by qRT-PCR. Average fold-changes detected between S and B *Aedes aegypti* mosquitoes of the LVP, CTM and Rex-D strains by RNA-seq and qRT-PCR on a random selection of thirteen genes.

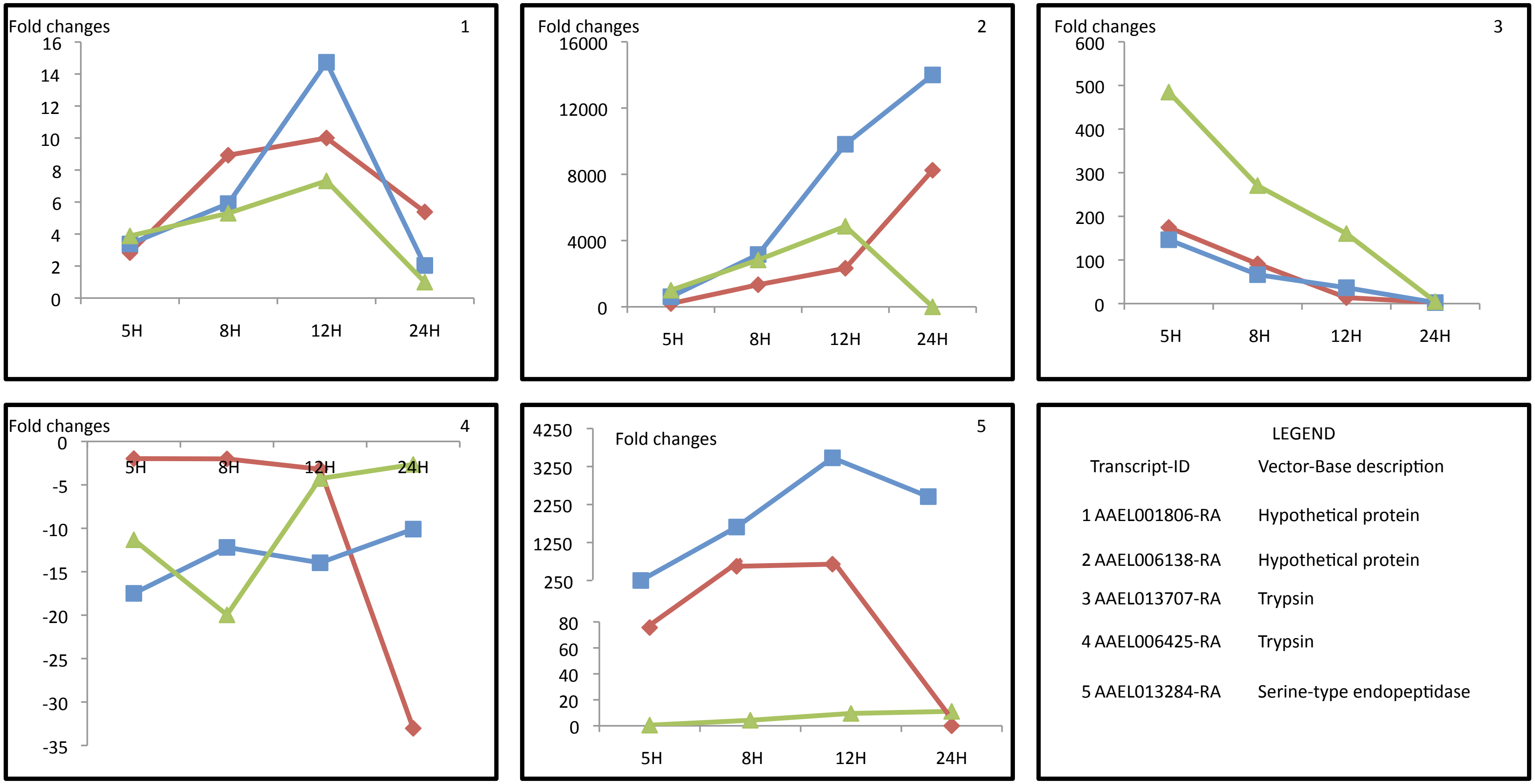

Figure S3 Expression profile of five transcripts between 5 and 24 hours PBM in three *Aedes aegypti* strains. Fold-changes between sugar and blood-fed mosquitoes at 5, 8, 12 and 24 h PBM, as assessed by q-RT PCR on five transcripts for mosquitoes of the LVP (red), CTM (blue) and Rex-D (green) strains.

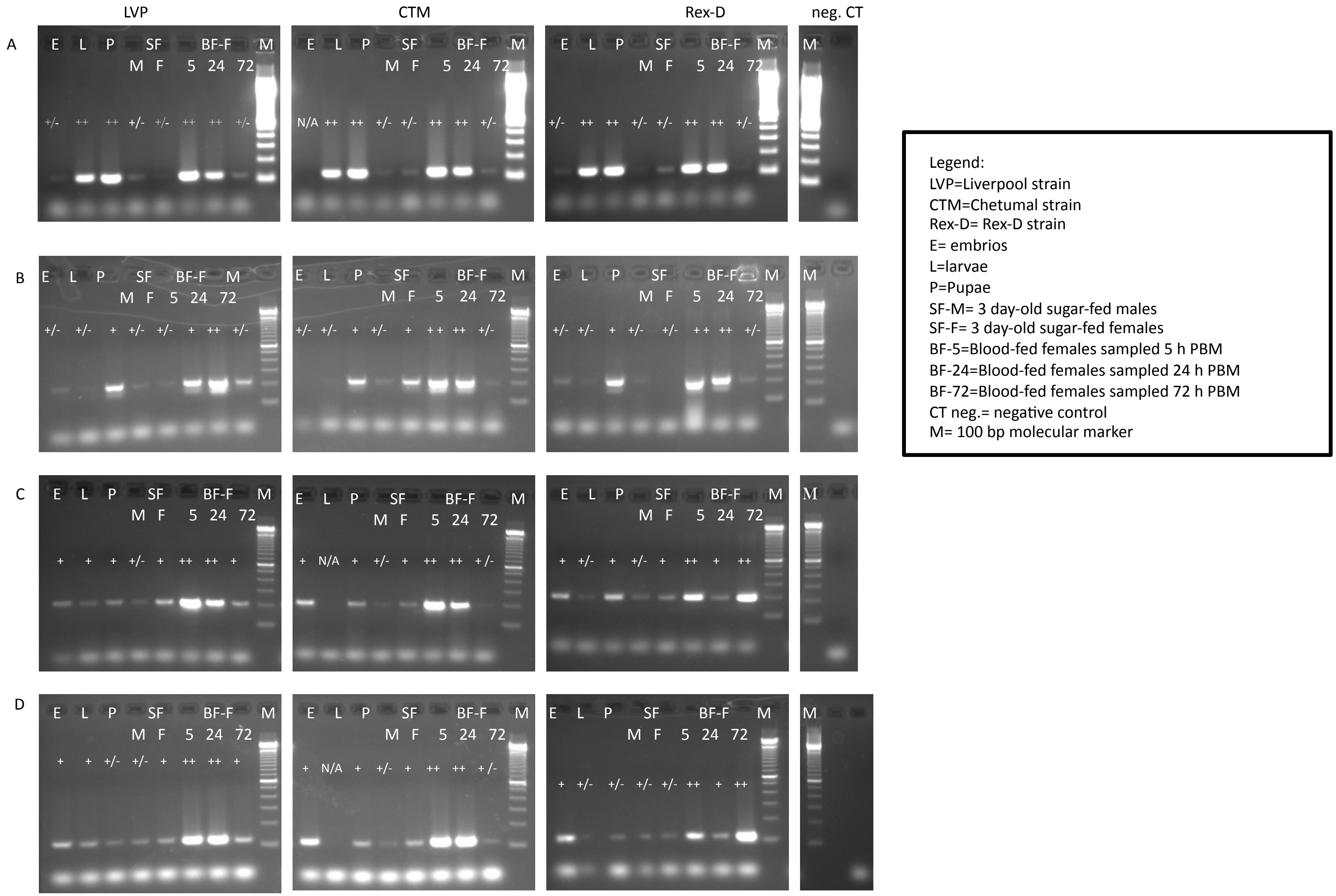

Figure S4 Results of RT-PCR on 8 developmental stages of three strains of *Ae. aegypti* mosquitoes for transcripts (A) AAEL013584-RA and (B) AAEL10196-RA, (C) AAEL013712-RA , (D) AAEL013713-RA showing the PCR bands classification as N/A, +/-, + or ++ .

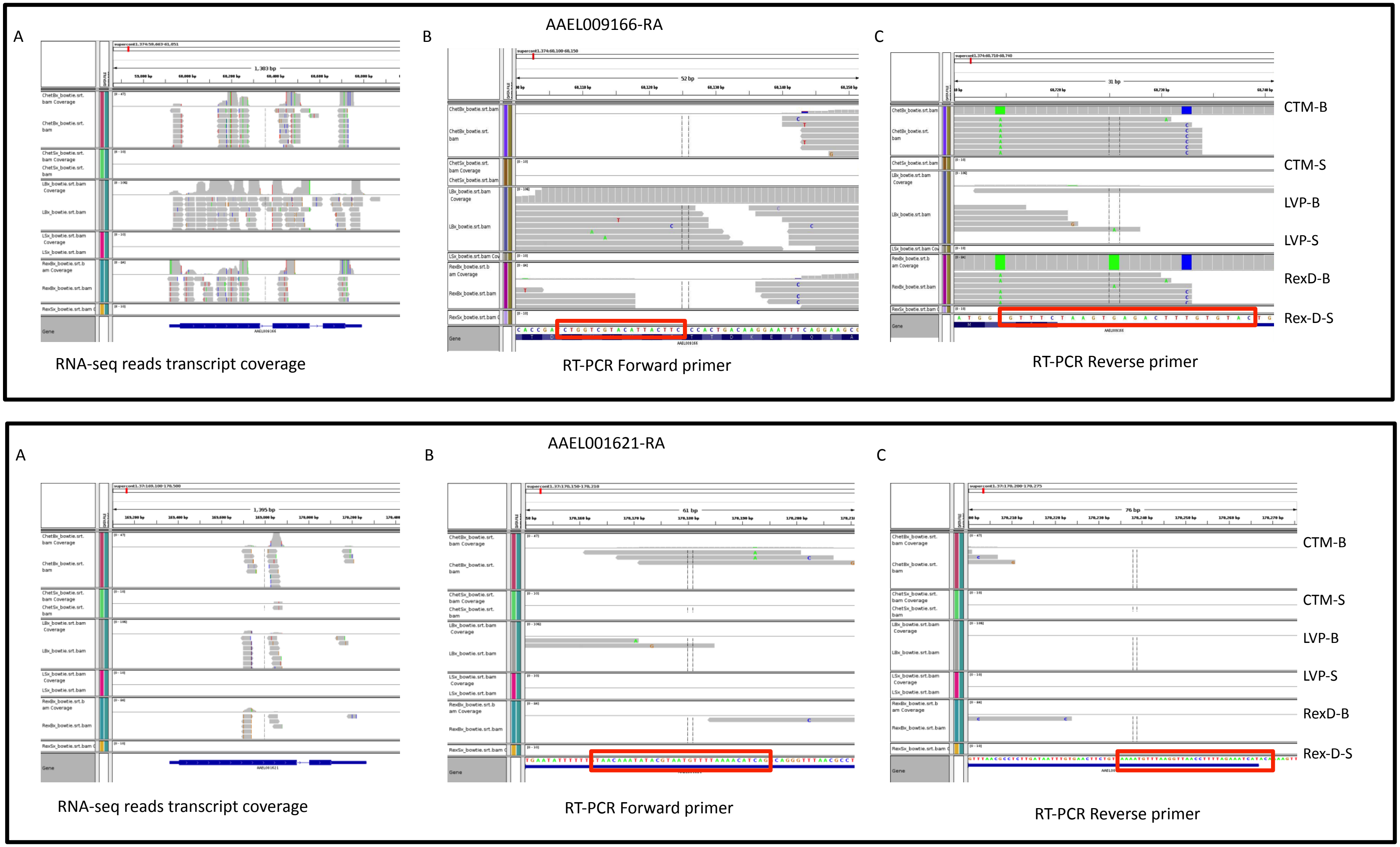

Figure S5 RNA-seq reads coverage (A) and RT-PCR primers position (B,C) in the three *Ae. aegytpi* strain tested for transcript AAEL009166-RA and AAEL001621-RA. In each read (gray raw), mismatches with respect to the reference *Ae. aegypti* genome sequence are shown in colors within the gray raw. RNA-seq reads were mapped to the *Ae. aegypti* genome reference sequence requiring unique match with a maximum of two mismatches. As a consequence, the absence of coverage along a transcript can be interpreted as not-unique sequence within the *Ae. aegypti* genome and/or sequence with high variation (i.e. more than 2 mismatches, insertions, deletions).

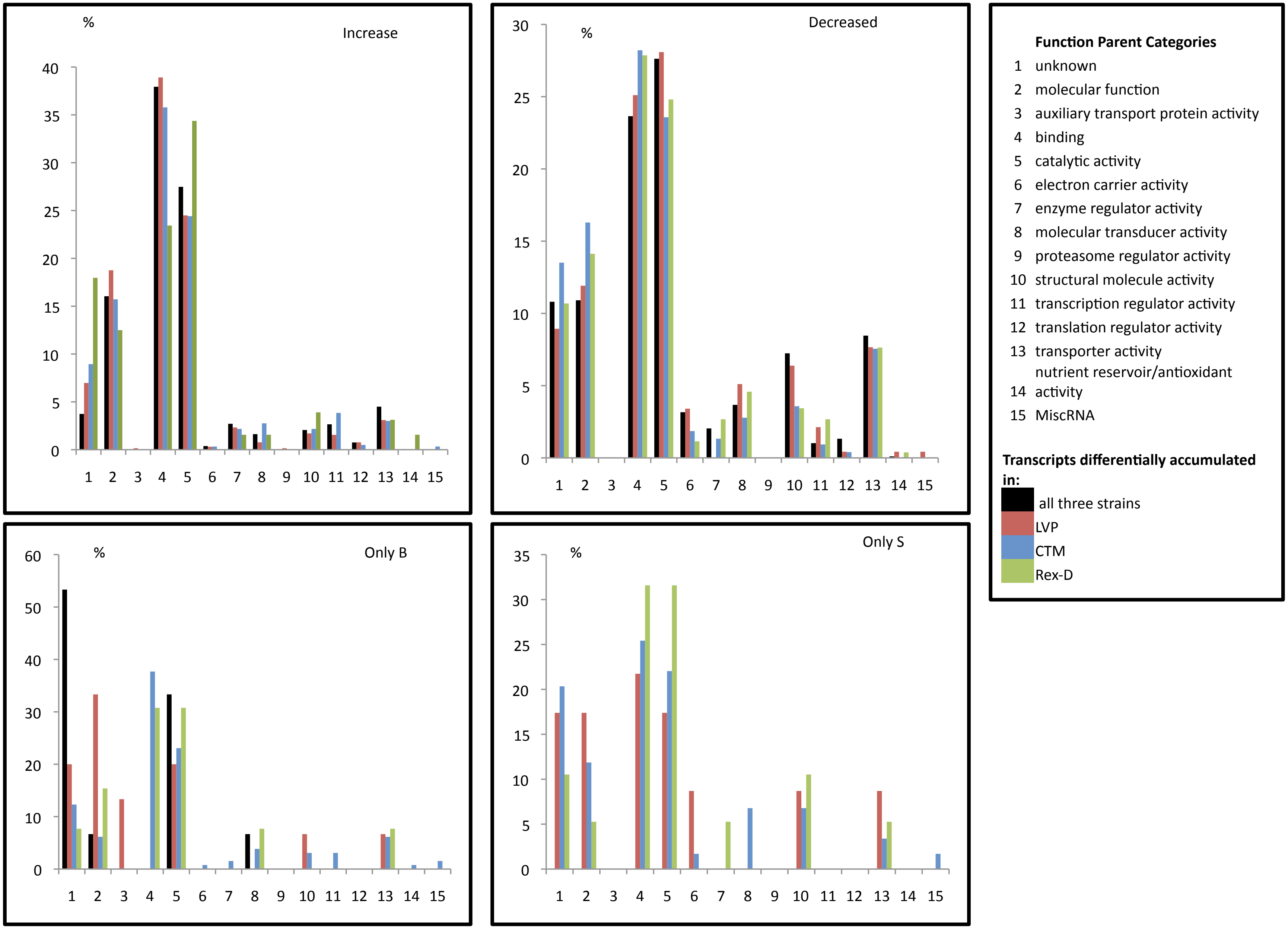

Figure S6 Function parent of differentially-accumulated transcripts. Function parent attribution of the transcripts accumulated differentially at 5 hPBM mosquitoes of the LVP, CTM and Rex-D strains.

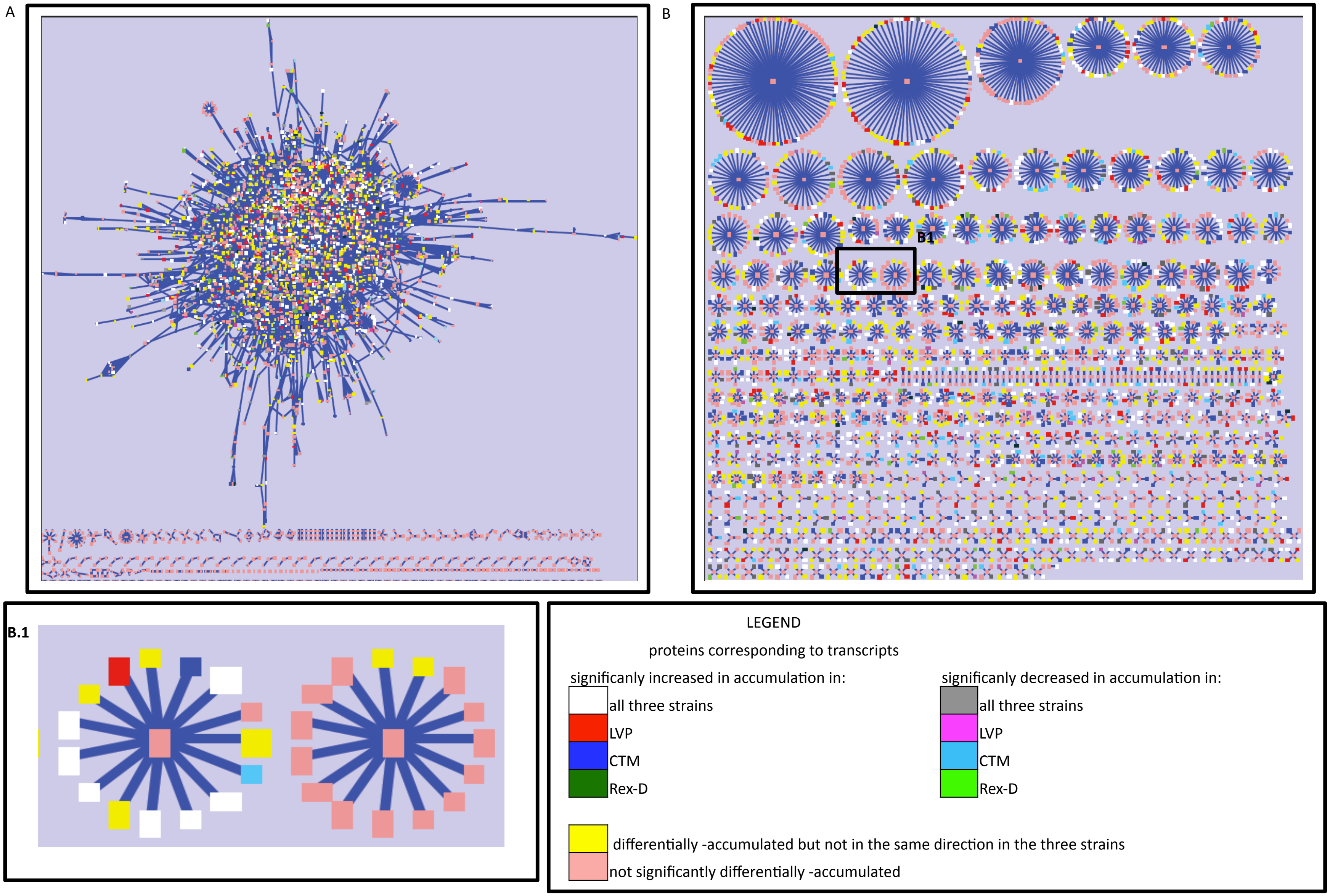

Figure S7 Protein network. *Aedes aegypti* protein network (A) and derived functional modules (B) (Guo *et al*., 2010). Proteins corresponding to transcripts identified by RNA-seq as accumulated differentially between B and S mosquitoes in three *Ae. aegypti* strains analyzed are in different colors. Examples of modules with an enrichment, or exclusive presence ,of proteins corresponding to transcripts either not responsive or differentially accumulated 5hPBM are show in panel B1.

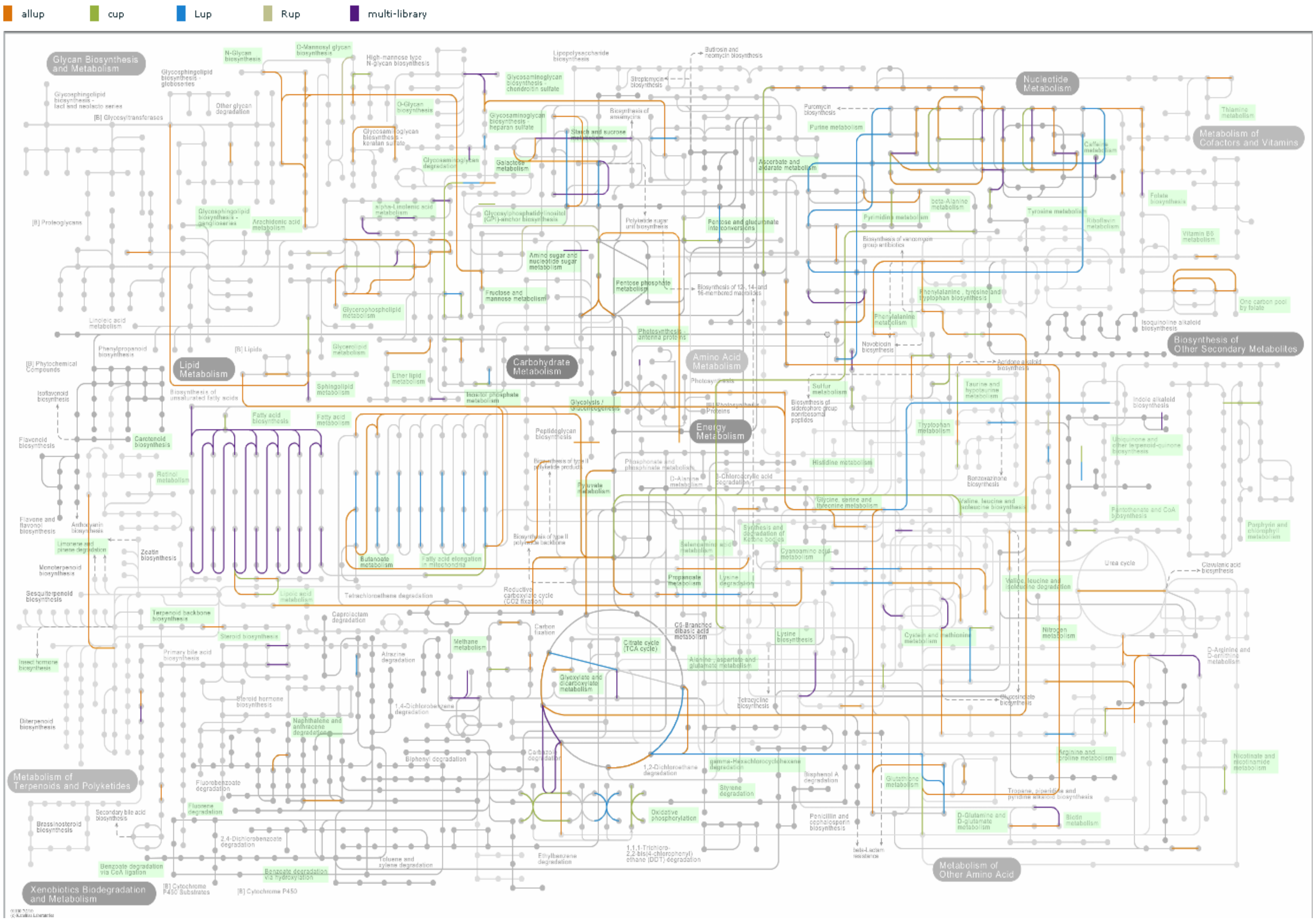

Figure S8 Metabolic pathways corresponding to transcripts increased in accumulation 5hPBM in all three strains (allup), in LVP (Lup), in CTM (cup) and in Rex-D (Rup) are visualized by LinkinPath (Ingsriswang et al., 2011). Multi-library corresponds to pathways elicited by transcripts increased in accumulation in more than one tested condition.

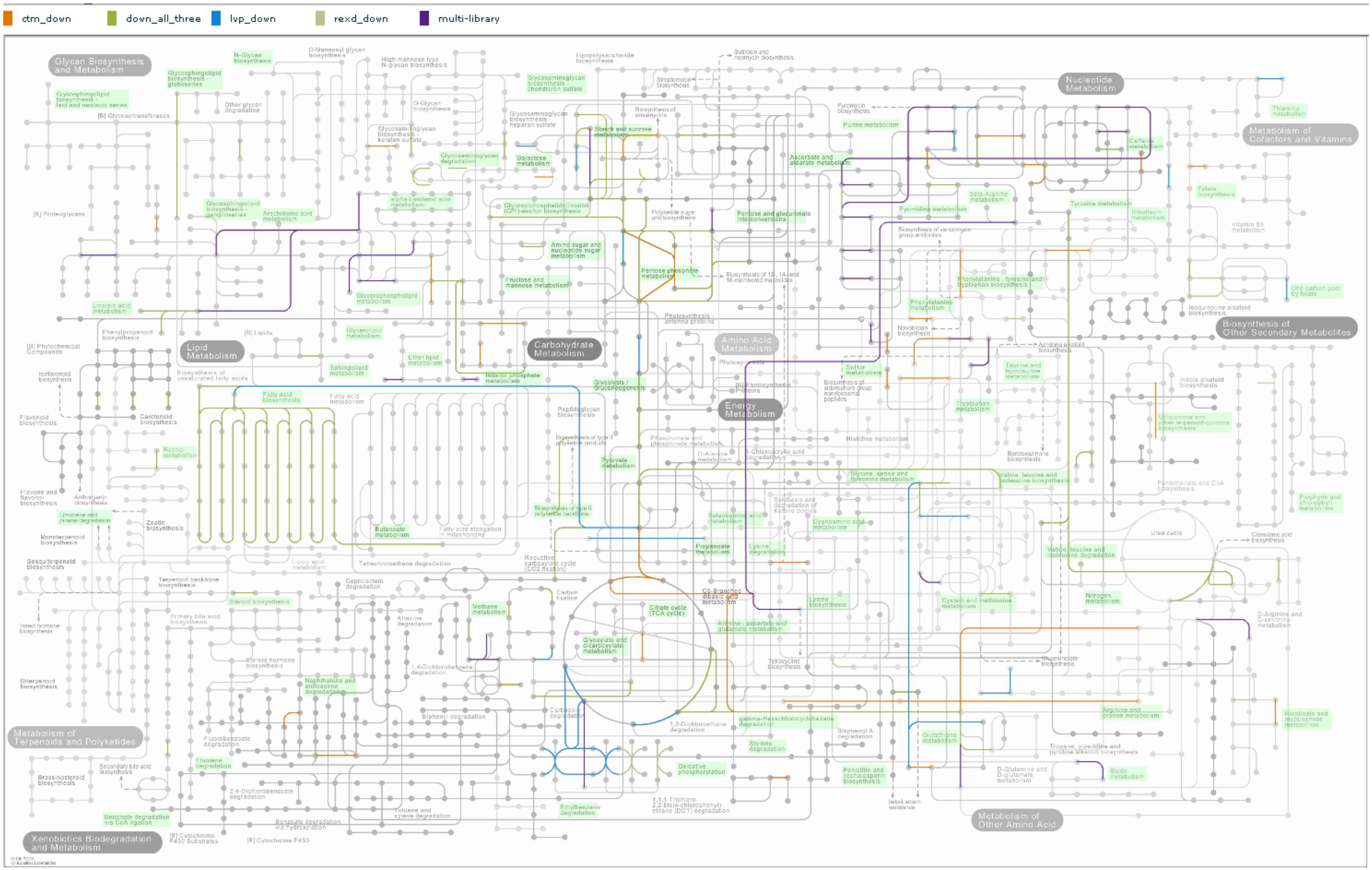

Figure S9 Metabolic pathways corresponding to transcripts decreased in accumulation 5hPBM in all three strains (down_all_three), in LVP (lvp_down), in CTM (ctm_down) and in Rex-D (rexd_down) are visualized by LinkinPath (Ingsriswang et al., 2011). Multi-library corresponds to pathways elicited by transcripts increased in accumulation in more than one tested condition.

Table S1 RT-PCR primers and conditions.

Best match to PFAM database	Transcript ID	Forward	Reverse	TA	ET	N. cyc.
Ins_allergen_rp	AAEL013127-RB	CTACTTAACCACTGACAAGGAATTTC	ATGAGATAGCACAAAAAAATTAGAGGTTA	60C	30 sec	25
	AAEL013584-RA	CATCAGATACATCACAATCGATCAAAGAACC	CCTTCAGGGTGCGTGTGTCTG	68C	30 sec	30
	AAEL013577-RA	TCCATCAGCAAGTAGATCTACAACAC	AAGTCATCCTTCAGGGCACG	62C	30 sec	30
	AAEL013577-RB	TCCATCAGCAAGTAGATCTACAACAC	AAGTCATCCTTCAGGGCACG	62C	30 sec	30
	AAEL010431-RA	TCCATCAGCAAGTAGATCTACAACAC	AAGTCATCCTTCAGGGCACG	62C	30 sec	30
	AAEL010429-RA	TCCATCAGCAAGTAGATCTACAACAC	AAGTCATCCTTCAGGGCACG	62C	30 sec	30
	AAEL009166-RA	TCTGGTCGTACATTACTTCA	GTACACAAAGTCTCACTTAGAAAC	54C	45 sec	25
	AAEL013118-RA	TTTTATGATTTATGTGTACTGACATATTC	TTTTATAACTGGTTTTTAGTATCCCC	57C	30 sec	35
	AAEL001621-RA	GTAACAAATATACGTAATGTTTTAAAACATCAG	GTATGATTTCTAAAAGGTTAACCTTAAACATTT	60C	30 sec	30
Peptidase_C1	AAEL015312-RA	CACAGCACGTAGAGATGTTCC	CCTGGCTCCAAAGTAGGACTGG	60C	30 sec	30
	AAEL012216-RA	TGCACAGCACAAATAACCCCC	CCTGGCTCCAAAGTAGGACTGG	65C	30 sec	30
	AAEL007585-RA	TGCACAGCACAAATAACCCCC	CCTGGCTCCAAAGTAGGACTGG	65C	30 sec	30
Trypsin	AAEL013713-RA	CCACTGACTTCGGCCAC	GGCAATACCCTGGTCGGAGTT	62	30 sec	30
	AAEL013712-RA	CGTTGAGCGGGGTTGGA	CATAGTCGGTGGTAACTTCATCGAT	62	30 sec	30
	AAEL010196-RA	*ACTCTTGCCAGGGTGATTCT	*TATTTTTAATCATTTCATTA	45C	30 sec	30
others	AAEL013706-RA	CCACCGACTACGATTTTGCG	GGAGAACTTCACGATTCAGCTGG	65C	30 sec	30
Vitellogenin_N	AAEL006126-RB	GATCAAGCTTCACCAGTGTCTTACAG	CATACTCTGGCTGCTTGACGTAG	63C	45 sec	30
	AAEL002908-RA	CGGAGACTACTGAGCAGTTATGGG	TCTAATGTAACTTTCAGACATTTTCG	59C	30 sec	35

*from Brackney *et al.*, 2010.

Primer pairs and gene amplification conditions (Annealing temperature=TA, extension time=ET, number of cycles=N.cyc) used to assay the abundance of the listed transcripts during *Ae. aegypti* mosquito development.

Table S2 List of transcripts accumulated differentially less than 2- fold and not accumulated differentially between sugar-fed mosquitoes across strains and between sugar- and blood-fed mosquitoes within each strain.

Table S3 Differential accumulation of transcripts among sugar-fed mosquitoes. Actual N. of reads detected and normalized log2 fold changes in accumulation (FC) for the transcripts significantly accumulated differentially between sugar-fed mosquitoes of the LVP, CTM and Rex-D strains. NA stands for not determined.

Table S4 Mosquito weight and blood-meal.

Table S5 Actual Number of reads and normalized log2 fold-changes in accumulation of transcripts between blood- and sugar-fed mosquitoes of the *Aedes aegypti* LVP, CTM and Rex-D strains. NA=not detected; F= not differentially accumulated at p<0.001; T=differentially accumulated at p<0.001.

Tables S2 –S5 are available for download at http://www.g3journal.org/lookup/suppl/doi:10.1534/g3.111.001107/-/DC1 as Excel files.

Table S6 Average fold changes of a random selection of thirteen genes in sugar- (S) and blood-fed (B) *Ae. aegypti* mosquitoes of three different strains as detected by qPCR.

		CTM			
transcript-ID	Function descriptors	B (std)	S (std)	average fold changes	t test
AAEL006138-RA	Vitellogenin-A1	1.57 (0.93)	1.4E-3 (3.21E-4)	10.1	t=3.29 (df=11) p=0.007
AAEL013284-RA	Serine-type endopeptidase AaLT	0.44 (0.16)	2.02 E-3 (1.08E-3)	7.75	t=5.44 (df=9) p<0.001
AAEL013707-RA	Trypsin-1	0.59 (0.52)	7.11E-3 (8.26 E-3)	6.39	t=2.58 (df=10) p=0.027
AAEL001806-RA	Lipid binding	0.21 (0.12)	2.84E-2 (2.40 E-3)	2.88	t=2.93 (df=7) p=0.022
AAEL014734-RA	Catalytic activity	0.16 (7.96 E-2)	1.80 E-2 (9.00 E-3)	3.19	t=3.60 (df=7) p=0.009
AAEL011470-RA	Protein binding	6.34E-2 (2.93 E-2)	4.72E-2 (1.82E-2)	0.42	t=1.23 (df=7) p=0.340
AAEL013005-RA	Molecular function	1.69E-2 (5.43E-3)	1.42E-2 (4E-3)	0.42	t=0.81 (df=6) p=0.451
AAEL002565-RA	Structural constituent of Cytoskeleton	7.38E-2(4.70E-2)	0.15(0.10)	-1.03	t=-1.82 (df=11) p=0.096
AAEL008848-RA	Catalytic activity	0.39 (0.24)	0.55 (0.33)	-0.51	t=-1.02 (df=11) p=0.328
AAEL012175-RA	Catalytic activity	0.59 (0.58)	1.16 (0.83)	-0.98	t=-1.52 (df=12) p=0.153
AAEL011871-RA	Electron Transporter	7.6E-2 (0.11)	9.25E-2 (9.17E-2)	6.6	t=-0.27 (df=9) p=0.792
AAEL006425-RA	Trypsin	8.25E-2 (8.18 E-2)	0.83 (0.15)	-3.33	t=-9.59 (df=8) p<0.001
AAEL008701-RA	Iron Ion Binding	1.07E-3 (5.10E-4)	1.90E-2 (6.91E-3)	-4.15	t=-6.39(df=9) p<0.001

		Rex-D			
**transcript-ID**	**Function descriptors**	B (std)	S (std)	average fold changes	t test
AAEL006138-RA	Vitellogenin-A1	2.69 (0.91)	2E-2 (2.95E-2)	7.05	t=6.53 (df=8) p<0.001
AAEL013284-RA	Serine-type endopeptidase AaLT	1.25 (0.36)	2.38E-2 (5.55 E-2)	5.71	t=8.30 (df=9) p<0.001
AAEL013707-RA	Trypsin-1	0.41 (7.50E-2)	1.49 E-2 (9.75 E-3)	4.78	t=10.45 (df=6) p<0.001
AAEL001806-RA	Lipid binding	0.17 (0.04)	2.63E-2 (7.23 E-3)	2.65	t=7.08 (df=7) p<0.001
AAEL014734-RA	Catalytic activity	3.88 E-2 (2.96 E-2)	4.75 E-3 (3.73 E-3)	3.03	t=2.87 (df=8) p=0.021
AAEL011470-RA	Protein binding	2.09 (2.34E-2)	2.13 (0.16)	-0.03	t=-0.60 (df=9) p=0.566
AAEL013005-RA	Molecular function	1.80E-2 (4.29E-3)	7.11E-3(2.26E-4)	1.34	t=5.08 (df=6) p=0.002
AAEL002565-RA	Structural constituent of Cytoskeleton	6.75E-2(1.11E-2)	7.32E-2(2.88E-2)	-0.12	t=-0.37 (df=6) p=0.724
AAEL008848-RA	Catalytic activity	0.21 (0.02)	0.28(1.54)	0.62	t=0.71 (df=8) p=0.503
AAEL012175-RA	Catalytic activity	0.37 (0.25)	0.77 (0.38)	-3.34	t=-2.01 (df=9) p=0.076
AAEL011871-RA	Electron Transporter	0.45 (0.13)	0.69 (0.44)	-0.62	t=-1.19(df=9) p=0.266
AAEL006425-RA	Trypsin	2.84E-2 (4.00E-2)	0.39 (0.17)	-3.78	t=-4.53(df=8) p=0.002
AAEL008701-RA	Iron Ion Binding	1.89E-3 (2.11E-3)	1.91E-2 (5.99E-3)	-3.34	t=-4.22(df=6) p=0.006

Results for LTV are as previously reported (Bonizzoni *et al*., 2011).

Table S7 Strain-signature. List of transcripts differentially accumulated (UP=increased; DOWN=decreased) at 5 hPBM.

Table S7 is available for download at http://www.g3journal.org/lookup/suppl/doi:10.1534/g3.111.001107/-/DC1 as an Excel file.

Table S8 Fold-changes between blood- and sugar-fed *Ae. aegypti* mosquitoes of three different strains as detected by qPCR at 5, 8, 12 and 24 hPBM.

			LTV			
transcript ID	Function descriptors		B (std)	S (std)	average fold-changes	
					Actual	Log2
AAEL001806-RA	Lipid binding	5 hPBM	0.14 (3.30E-2)	5.05E-2 (1.36E-2)	2.84	1.50589093
		8 hPBM	0.45 (9.40E-2)	5.05E-2 (1.36E-2)	8.93	3.158660175
		12 hPBM	0.50 (0.11)	5.05E-2 (1.36E-2)	10.01	3.323370069
		24 hPBM	0.27 (5.20E-2)	5.05E-2 (1.36E-2)	5.38	2.427606173
AAEL006138-RA	Vitellogenin-A1	5 hPBM	1.40 (0.86)	7.08e-3 (1.0E-2)	197.84	7.628190335
		8 hPBM	9.44 (2.66)	7.08e-3 (1.0E-2)	1332.6	10.38002808
		12 hPBM	16.49 (3.99)	7.08e-3 (1.0E-2)	2328.64	11.18527191
		24 hPBM	58.39 (21.13)	7.08e-3 (1.0E-2)	8246.53	13.00957147
AAEL013707-RA	Trypsin-1	5 hPBM	3.05 (0.66)	1.75E-2(8.66E-3)	174.52	7.447248569
		8 hPBM	1.59 (1.29)	1.75E-2(8.66E-3)	90.96	6.507160349
		12 hPBM	0.29 (7.04E-2)	1.75E-2(8.66E-3)	13.64	3.769771739
		24 hPBM	4.82E-2 (5.3E-2)	1.75E-2(8.66E-3)	2.75	1.459431619
AAEL006425-RA	Trypsin	5 hPBM	7.65E-2 (2.48E-2)	0.15 (5.20E-2)	0.51	-0.971430848
		8 hPBM	7.52 E-2 (3.0 E-2)	0.15 (5.20E-2)	0.5	-1
		12 hPBM	4.73 E-2 (5.22E-3)	0.15 (5.20E-2)	0.31	-1.689659879
		24 hPBM	0.18 (7.48E-2)	0.15 (5.20E-2)	0.03	-5.058893689
AAEL013284-RA	Serine-type endopeptidase AaLT	5 hPBM	0.49 (0.25)	6.47E-3 (4.18E-3)	75.65	6.241268177
		8 hPBM	4.04 (0.72)	6.47E-3 (4.18E-3)	269.58	8.074569657
		12 hPBM	4.40 (0.72)	6.47E-3 (4.18E-3)	173.47	7.438542374
		24 hPBM	1.72 (1.01)	6.47E-3 (4.18E-3)	2.42E-04	-12.02467797

transcript ID	Function descriptors		CTM			
			B (std)	S (std)	average fold-changes	
					Actual	Log2
AAEL001806-RA	Lipid binding	5 hPBM	0.12 (2.31E-2)	3.41E-2 (9.63 E-3)	3.38	1.757023247
		8 hPBM	0.20 (3.17E-2)	3.41E-2 (9.63 E-3)	5.9	2.560714954
		12 hPBM	0.50 (0.18)	3.41E-2 (9.63 E-3)	14.73	3.880685525
		24 hPBM	6.94E-2 (3.25E-2)	3.41E-2 (9.63 E-3)	2.04	1.028569152
AAEL006138-RA	Vitellogenin-A1	5 hPBM	1.54 (0.76)	1.4E-3 (3.21E-4)	609.14	9.250630034
		8 hPBM	8.00 (1.41)	1.4E-3 (3.21E-4)	3158.14	11.62485941
		12 hPBM	24.87 (7.70)	1.4E-3 (3.21E-4)	9814.12	13.2606432
		24 hPBM	35.48 (5.24)	1.4E-3 (3.21E-4)	13999	13.77303512
AAEL013707-RA	Trypsin-1	5 hPBM	0.72 (0.37)	4.91E-3 (1.15 E-3)	143.32	7.163096138
		8 hPBM	0.33 (8.36E-2)	4.91E-3 (1.15 E-3)	66.29	6.050719348
		12 hPBM	0.179 (5.79E-2)	4.91E-3 (1.15 E-3)	36.42	5.186659017
		24 hPBM	1.13E-2 (7.78E-3)	4.91E-3 (1.15 E-3)	2.3	1.201633861
AAEL006425-RA	Trypsin	5 hPBM	2.67E-2 (1.21E-2)	0.47 (0.13)	0.057	-4.13289427
		8 hPBM	3.82 E-2 (3.89E-2)	0.47 (0.13)	0.08	-3.64385619
		12 hPBM	3.33 E-2 (2.02E-2)	0.47 (0.13)	0.07	-3.836501268
		24 hPBM	6.95E-2 (3.24E-2)	0.47 (0.13)	0.1	-3.321928095
AAEL013284-RA	Serine-type endopeptidase AaLT	5 hPBM	0.28 (0.11)	1.14 E-3 (129E-3)	250.45	7.968378801
		8 hPBM	1.88 (0.54)	1.14 E-3 (129E-3)	1657.58	10.69486279
		12 hPBM	3.95 (1.30)	1.14 E-3 (129E-3)	3479.47	11.76465185
		24 hPBM	2.79 (1.03)	1.14 E-3 (129E-3)	2458.05	11.26329855

transcript ID	Function descriptors		Rex-D			
			B (std)	S (std)	average fold-changes	
					actual	Log2
AAEL001806-RA	Lipid binding	5 hPBM	0.13 (1.36E-2)	3.47E-2 (1.08E-2)	3.88	1.956056652
		8 hPBM	0.18 (4.91E-2)	3.47E-2 (1.08E-2)	5.3	2.40599236
		12 hPBM	0.25 (0.14)	3.47E-2 (1.08E-2)	7.32	2.871843649
		24 hPBM	3.45E-2 (1.09E-2)	3.47E-2 (1.08E-2)	0.99	-0.01449957
AAEL006138-RA	Vitellogenin-A1	5 hPBM	1.53 (0.30)	1.53E-3 (7.98E-4)	1000.65	9.966721732
		8 hPBM	4.33 (1.75)	1.53E-3 (7.98E-4)	2829.58	11.46637221
		12 hPBM	7.44 (0.88)	1.53E-3 (7.98E-4)	4863.71	12.24784149
		24 hPBM	1.53E-3 (8.03E-4)	1.53E-3 (7.98E-4)	1.001	0.001441974
AAEL013707-RA	Trypsin-1	5 hPBM	1.35 (8.72E-2)	2.79 E-3 (5.40E-4)	484.74	8.921067326
		8 hPBM	0.72 (0.21)	2.79 E-3 (5.40E-4)	270.42	8.079058046
		12 hPBM	0.45 (0.26)	2.79 E-3 (5.40E-4)	160.5	7.326429487
		24 hPBM	1.26E-2 (1.12E-2)	2.79 E-3 (5.40E-4)	4.52	2.176322773
AAEL006425-RA	Trypsin	5 hPBM	3.07E-2 (9.94E-3)	0.35 (0.35)	0.09	-3.473931188
		8 hPBM	1.74 E-2 (5.09E-3)	0.35 (0.35)	0.05	-4.321928095
		12 hPBM	8.14E-2 (6.27E-2)	0.35 (0.35)	0.23	-2.120294234
		24 hPBM	0.13 (2.62E-2)	0.35 (0.35)	0.38	-1.395928676
AAEL013284-RA	Serine-type endopeptidase AaLT	5 hPBM	3.06E-4 (1.12E-4)	6.33E-4 (3.43 E-4)	0.48	-1.058893689
		8 hPBM	2.70E-3 (7.85E-4)	6.33E-4 (3.43 E-4)	4.27	2.09423607
		12 hPBM	6.04E-3 (4.44E-3)	6.33E-4 (3.43 E-4)	9.53	3.252476214
		24 hPBM	7.04E-3(5.54E-3)	6.33E-4 (3.43 E-4)	11.11	3.473786912

Table S9 Expression profile of eighteen *Ae. aegypti* transcripts during development. RT-PCR results of selected transcripts over 8 developmental stages of *Ae. aegypti* mosquitoes. RT-PCR results were classified in four groups as absence of amplification (N/A); faint amplification (+/-); amplification (+); strong amplification (++) (Figure S4).

Best match to PFAM database	transcript-ID (1)	strain	Developmental stages (2)							
Ins_allergen_rp			E	L	P	SF-M	SF-F	BF-5H	BF-24H	BF-72H
	AAEL013127-RB	LTV	N/A	N/A	N/A	N/A	N/A	+	+	N/A
		CTM	N/A	N/A	N/A	N/A	N/A	+	+/-	N/A
		Rex-D (3)	N/A	N/A	N/A	N/A	N/A	N/A	+	N/A
	AAEL013584-RA	LTV	+/-	++	++	+/-	+/-	++	++	+/-
		CTM	N/A	++	++	+/-	+/-	++	++	+/-
		Rex-D	+/-	++	++	+/-	+/-	++	++	+/-
	AAEL013577-RA,AAEL013577-RB,	LTV	N/A	N/A	++	+/-	+/-	++	++	+
	AAEL010431-RA, AAEL010429-RA	CTM	N/A	+/-	++	+	+	++	+/-	+/-
	(4)	Rex-D	+/-	N/A	++	+/-	++	++	++	+/-
	AAEL009166-RA	LTV	N/A	N/A	N/A	N/A	N/A	+	N/A	N/A
		CTM	N/A	N/A	N/A	N/A	N/A	N/A	N/A	N/A
		Rex-D	N/A	N/A	N/A	N/A	N/A	N/A	N/A	N/A
	AAEL013118-RA	LTV	N/A	N/A	N/A	N/A	N/A	+	N/A	N/A
		CTM	N/A	N/A	N/A	N/A	N/A	+	N/A	N/A
		Rex-D	N/A	N/A	N/A	N/A	N/A	+/-	N/A	N/A
	AAEL001621-RA	LTV	N/A	N/A	N/A	N/A	N/A	+	N/A	N/A
		CTM	N/A	N/A	N/A	N/A	N/A	N/A	N/A	N/A
		Rex-D	N/A	N/A	N/A	N/A	N/A	N/A	N/A	N/A
Peptidase_C1	AAEL015312-RA	LTV	+	+	+/-	+/-	+/-	++	++	++
		CTM	+/-	+/-	+	+	+/-	++	++	+
		Rex-D	+	+/-	+/-	+/-	+/-	+	++	+

Best match to PFAM database	transcript-ID (1)	strain	Developmental stages (2)							
	AAEL012216-RA, *AAEL007585-RA*	LTV	+/-	N/A	N/A	N/A	N/A	++	++	++
	(4)	CTM	+/-	+/-	+/-	++	+/-	++	++	++
		Rex-D	+/-	+/-	+/-	+/-	+	++	++	+
Trypsin	*AAEL013713-RA*	LTV	+	+	+/-	+/-	+	++	++	+
		CTM	+	N/A	+	+/-	+	++	++	+/-
		Rex-D	+	+/-	+/-	+/-	+/-	+	+	++
	AAEL013712-RA	LTV	+	+	+	+/-	+	++	++	+
		CTM	+	N/A	+	+/-	+	++	++	+/-
		Rex-D	+	+/-	+	+/-	+	++	+	++
	AAEL0010196-RA	LTV	+/-	+/-	+	+/-	+/-	+	++	+
		CTM	+/-	+/-	+	+/-	+	+	++	+/-
		Rex-D	+/-	+/-	+	+/-	+	+	++	+/-
unknown	AAEL013706-RA	LTV	+	N/A	+	+/-	+/-	++	++	++
		CTM	++	+	++	+	+	++	++	+
		Rex-D	+	+	++	+	+	++	++	+
	AAEL006126-RB	LTV	+	+	++	++	+	++	++	+
		CTM	+	++	++	++	+	++	++	+
		Rex-D	+	+	+	+	+	++	++	+
	AAEL002908-RA	LTV	+/-	+/-	+/-	+/-	+/-	++	++	++
		CTM	+/-	+/-	+/-	+/-	+/-	++	++	++
		Rex-D	+/-	+/-	+/-	+/-	+/-	++	++	++

(1)Transcripts significantly found only in B mosquitoes are in italics, (2) Developmental stages tested are: E=embryos, L=larvae, P=pupae, SF-M=sugar fed males, SF-F=sugar fed females, and blood fed females sampled 5, 24 and 72 hours post blood feeding for BF-5H, BF-24H and BF-72H; (3) when products of the PCR were used as template for a second PCR using forward primer 5' CAAGGAAGTGCTGAACTACTTGGAA and reverse primer 5'GCTTTTAAGCTCTTAACTCCTTTCG, a PCR band of expected size was detected in P, BF-5h, BF-24h; (4) Level of sequence identity among the four transcripts (AAEL013577-RA, AAEL013577-RB, AAEL010431-RA and AAEl010429-RA) and the two transcripts (AAEL012216-RA, AAEL007585-RA) prevented the design of transcript-specific primers.

Table S10 Comparison of number of transcripts from different functional classes. Fisher exact test for transcripts of different functional classes increased or decreased in accumulation 5 hBPM (A) consistently in all three strains, (B) in LVP, (C) in CTM (D) in Rex-D. Fisher exact test for transcripts of different functional classes (E) increased or (F) decreased in accumulation 5hBPH between LVP and CTM, LVP and Rex-D and CTM and Rex-D.

**A**	N. transcrips		
	Increased	Decreased	Fisher exact test two tailed p value
CS	59	68	<0.0001
DIV	268	75	<0.0001
PT	148	37	<0.0001
DM	9	7	0.4334
TMLCA	275	245	<0.0001
TRP	43	10	0.0186
UNK	265	143	0.6459
TTPMR	384	101	<0.001
STM	97	113	<0.001
**total**	**1548**	**799**	

**B**	N. transcripts		
	Increased	Decreased	Fisher exact test two tailed p value
CS	27	9	0.8495
DIV	77	25	0.4082
PT	50	15	0.388
DM	3	1	0.5816
TMLCA	77	56	0.0002
TRP	11	7	0.3001
UNK	102	31	0.1688
TTPMR	134	23	<0.001
STM	50	42	0.0002
**total**	**531**	**209**	

**C**	N. transcripts		
	Increased	Decreased	Fisher exact test two tailed p value
CS	62	44	0.0877
DIV	93	52	0.5196
PT	85	28	0.0488
DM	8	6	0.4072
TMLCA	159	135	<0.001
TRP	28	21	0.1232
UNK	183	100	0.487
TTPMR	281	86	<0.001
STM	135	50	0.0467
**total**	**1034**	**522**	

**D**	N. transcripts		
	Increased	Decreased	Fisher exact test two tailed p value
CS	3	16	0.1347
DIV	7	26	0.1732
PT	9	18	1
DM	0	2	1
TMLCA	35	50	0.0424
TRP	3	4	0.6848
UNK	22	31	0.1463
TTPMR	12	34	0.3957
STM	11	34	0.3013
**total**	**102**	**215**	

**E**	Fisher exact test two tailed p value		
	LVP/CTM	LVP/Rex-D	CTM/Rex-D
CS	0.4913	0.4524	0.2658
DIV	0.0014	0.0381	0.5838
PT	0.4472	1	0.8501
DM	0.7588	1	1
TMLCA	0.7092	0.0001	0.0001
TRP	0.4974	0.4813	0.7497
UNK	0.4892	0.5869	0.3449
TTPMR	0.432	0.0029	0.0005
STM	0.0385	0.7136	0.6416

**F**	Fisher exact test two tailed p value		
	LVP/CTM	LVP/Rex-D	CTM/Rex-D
CS	0.058	0.2166	0.7673
DIV	0.426	1	0.4295
PT	0.3846	0.7184	0.1332
DM	0.6797	1	1
TMLCA	0.8522	0.4332	0.5132
TRP	0.8318	0.3754	0.1804
UNK	0.2001	1	0.1384
TTPMR	0.0661	0.1568	0.9127
STM	0.002	0.2575	0.0212

Table S11 Immunity associated-transcripts. List of transcripts associated with immunity in *Ae. aegypti* with RNA-seq data (normalised fold change, FC) for the LVP, CTM and Rex-D strain. Significant fold-changes in accumulation between B and S mosquitoes are in bold. NA stands for not-detected. Transcript ID is as reported in VectorBase (Lawson et al., 2009) AAELXXXXXX-RX; AAEL is not shown for simplicity.

Table S11 is available for download at http://www.g3journal.org/lookup/suppl/doi:10.1534/g3.111.001107/-/DC1 as an Excel file.

Table S12 Enrichment of immunity-related classes of transcripts. P value associated with a hypergeometric test are shown for mosquitoes of the LVP, CTM and Rex-D strains. p values <0.01 are bold, p values <0.05 are in italicus.

Immunity-related classes	LVP	CTM	Rex-D
1,3-BETA-d Glucan Binding Proteins	0.421205879	0.093204	0.068521
Anti-microbial peptides	0.575258861	0.054398	0.133909
Autophagy Genes	*0.025244527*	*0.032705*	0.10294
Caspase Activator	1	0.636229	1
Caspases	0.596803387	*0.01438*	0.967236
Catalase	0.10875457	0.157481	0.434227
CLIP-domain serine protease	0.665377639	0.624128	0.068302
C-Type Lectins	0.939621148	0.961407	0.773125
Fibrinogen related protein (FREP)	0.729579204	0.963194	0.325004
Galectins	0.596803387	0.767986	0.34444
IMD Pathway members	0.799110015	0.678041	0.627499
inhibitors of apoptosis (IAP)	*0.040252257*	0.259983	*0.047204*
JAKSTAT Signal transduction	0.698987823	0.062487	0.153783
lysozyme	0.421205879	0.573253	0.239038
MD2-like proteins (ML)	0.976981595	0.730233	0.561666
others	*0.016960761*	0.039506	0.165959
peptidoglycan recognition protein	0.139416573	0.095835	0.161615
Peroxidase	*0.01692638*	*0.011764*	**0.000254**
prophenoloxidase	0.979316144	0.971402	0.758062
Relish-like proteins	0.254545294	0.347469	0.153783
Scavanger Receptors	0.086148092	**0.000283**	**0.000854**
Serine protease inhibitors	0.552011966	0.090862	0.100341
Small Regulatory RNA pathway members	**0.000923729**	*0.011925*	*0.033746*
spaetzle like (SPZ like)	0.851962443	0.095835	0.694443
Superoxido-dismutase	0.523084665	0.678041	0.110766
Thio-Ester containing proteins (TEP)	0.167866594	0.837272	0.239038
Toll Receptors/Pathway	0.22889149	0.46441	0.24858

Table S13 Proteins in clusters from the high-confidence protein interaction network corresponding to the transcripts identified as accumulated differentially between B and S mosquitoes by RNA-seq data in at least one of the three *Ae. aegypti* strains analyzed.

Table S14 Protein cluster enrichments. P values associated with a hypergeometric test are shown for protein clusters from the *Ae. aegypti* protein network comprising transcripts accumulated differentially at 5 hPBM. Significant (at $p < 0.05$) values are in color. N. T. is the number of transcripts detected as accumulated differentially by RNA-seq (transcripts found significantly accumulated only in B and S mosquitoes are included with transcripts increased and decreased in accumulation, respectively). N P. is the number of corresponding proteins identified in the protein network .

Table S13 and S14 are available for download at http://www.g3journal.org/lookup/suppl/doi:10.1534/g3.111.001107/-/DC1 as Excel files.
