# Supplementary figures and images for: Strain Variation in the Transcriptome of the Dengue Fever Vector, Aedes aegypti
Source: G3 (Bethesda). 2012 Jan 1;2(1):103–14. doi: 10.1534/g3.111.001107 (PMC3276191; doi:10.1534/g3.111.001107)

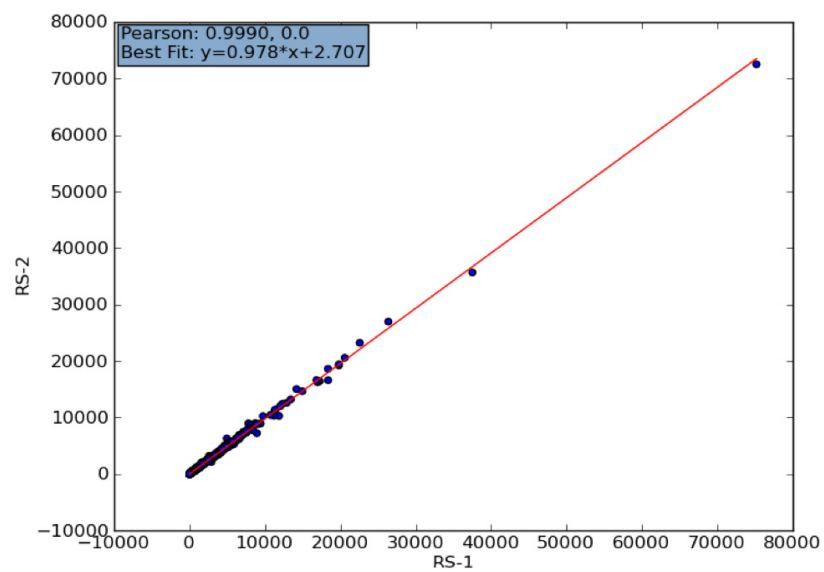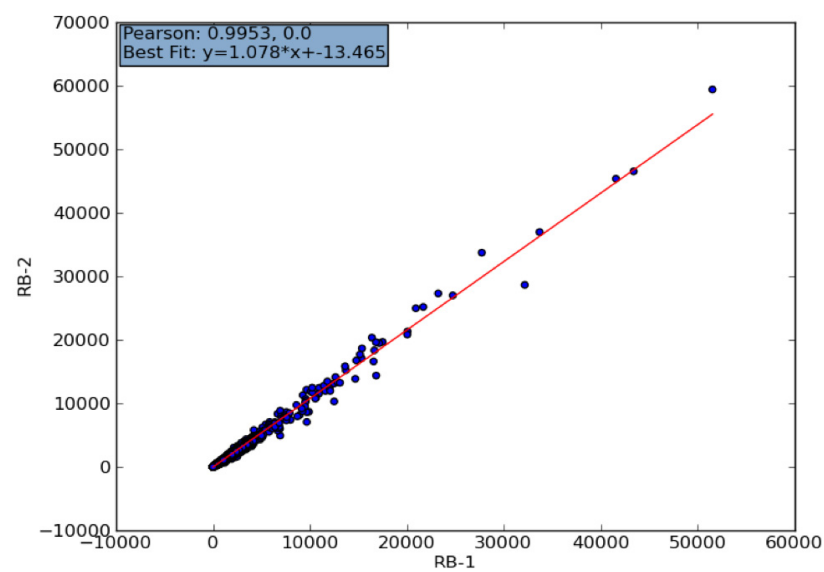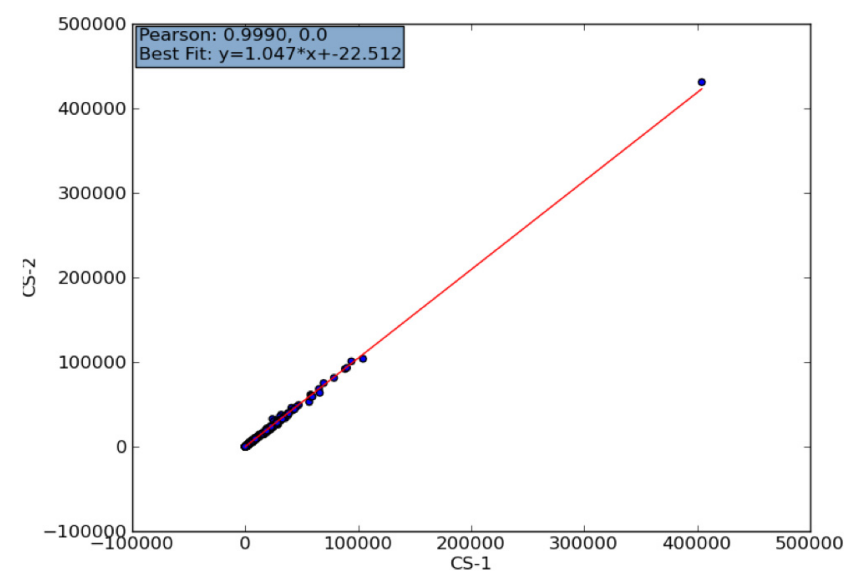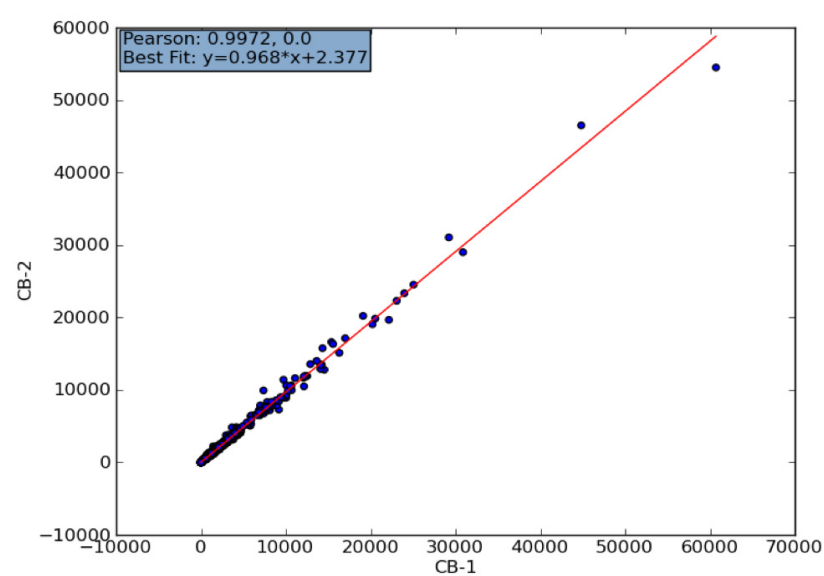

**Figure S1** Pearson correlation comparisons for RNA-seq replicates.

Supplement: Supporting Information [file supp_2.1.103_FigureS1.pdf]
