# Supplementary material for: Strain Variation in the Transcriptome of the Dengue Fever Vector, Aedes aegypti
Source: G3 (Bethesda). 2012 Jan 1;2(1):103–14. doi: 10.1534/g3.111.001107 (PMC3276191; doi:10.1534/g3.111.001107)
Supplement: Supporting Information [file supp_2.1.103_FigureS2.pdf]

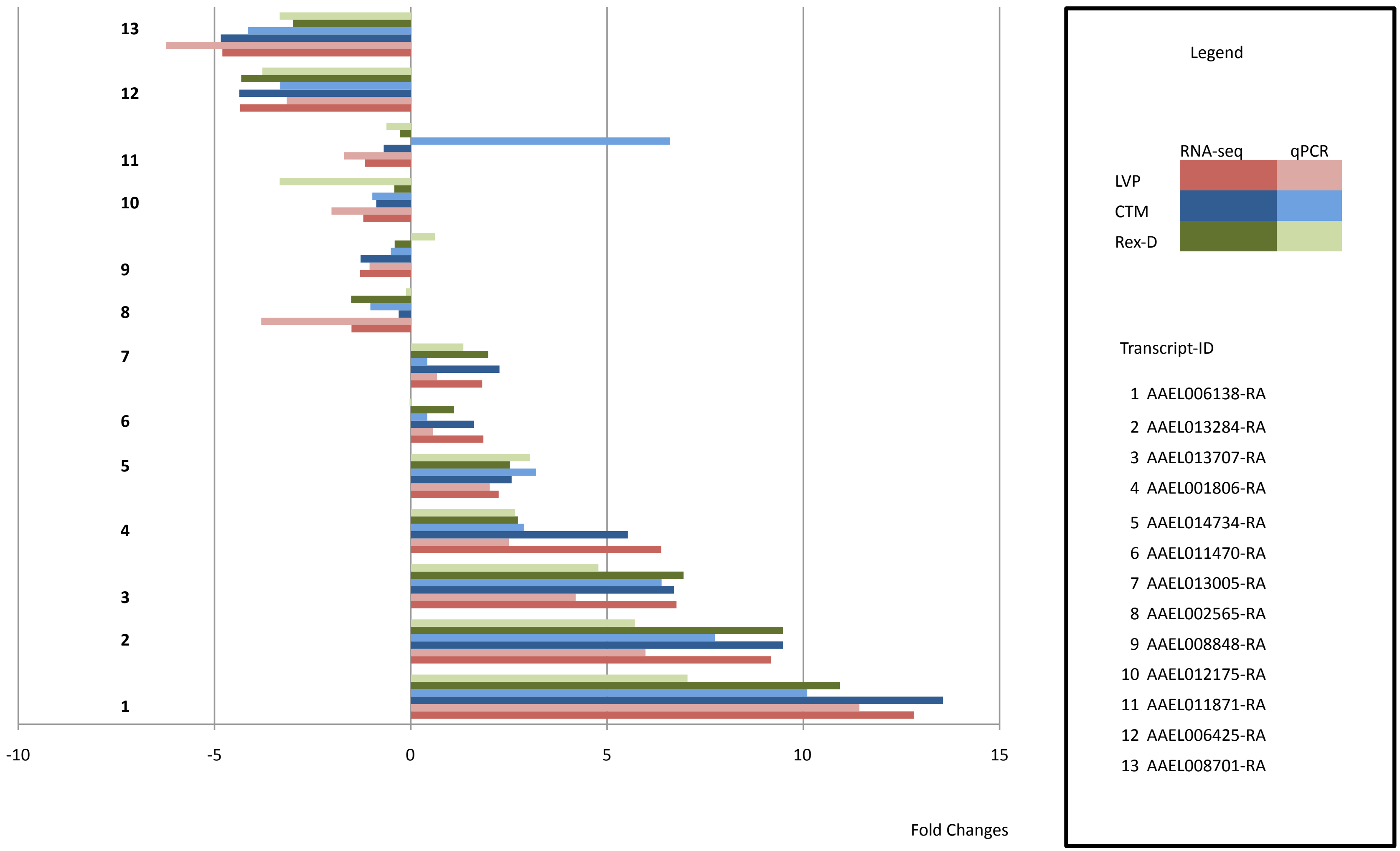

Figure S2 RNA-seq data validation by qRT-PCR. Average fold-changes detected between S and B *Aedes aegypti* mosquitoes of the LVP, CTM and Rex-D strains by RNA-seq and qRT-PCR on a random selection of thirteen genes.
