# Supplementary material for: Strain Variation in the Transcriptome of the Dengue Fever Vector, Aedes aegypti
Source: G3 (Bethesda). 2012 Jan 1;2(1):103–14. doi: 10.1534/g3.111.001107 (PMC3276191; doi:10.1534/g3.111.001107)
Supplement: Supporting Information [file supp_2.1.103_FigureS3.pdf]

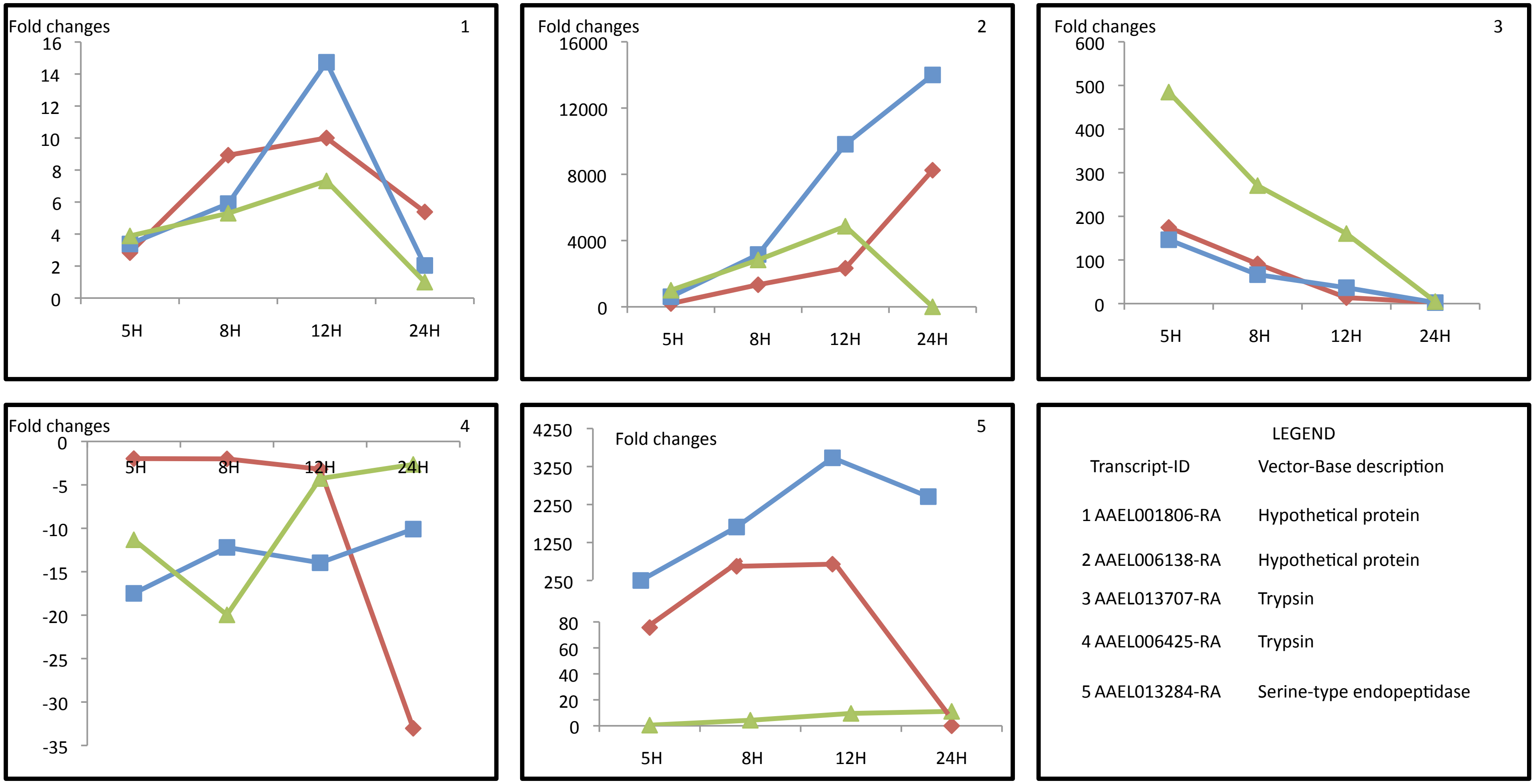

Figure S3 Expression profile of five transcripts between 5 and 24 hours PBM in three *Aedes aegypti* strains. Fold-changes between sugar and blood-fed mosquitoes at 5, 8, 12 and 24 h PBM, as assessed by q-RT PCR on five transcripts for mosquitoes of the LVP (red), CTM (blue) and Rex-D (green) strains.
