# Supplementary material for: Strain Variation in the Transcriptome of the Dengue Fever Vector, Aedes aegypti
Source: G3 (Bethesda). 2012 Jan 1;2(1):103–14. doi: 10.1534/g3.111.001107 (PMC3276191; doi:10.1534/g3.111.001107)
Supplement: Supporting Information [file supp_2.1.103_FigureS4.pdf]

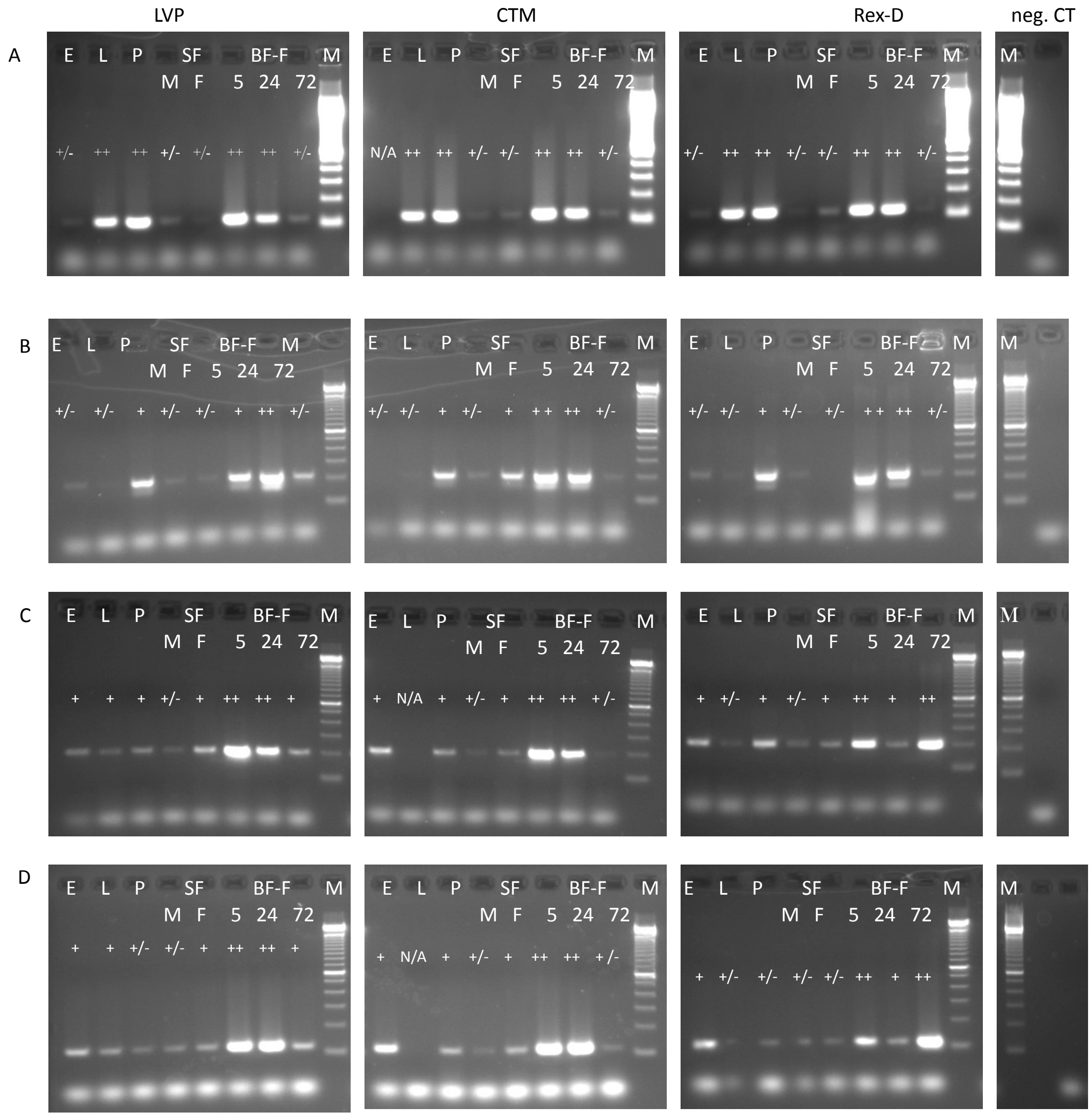

Legend:
LVP=Liverpool strain
CTM=Chetumal strain
Rex-D= Rex-D strain
E= embrios
L=larvae
P=Pupae
SF-M= 3 day-old sugar-fed males
SF-F= 3 day-old sugar-fed females
BF-5=Blood-fed females sampled 5 h PBM
BF-24=Blood-fed females sampled 24 h PBM
BF-72=Blood-fed females sampled 72 h PBM
CT neg.= negative control
M= 100 bp molecular marker

Figure S4 Results of RT-PCR on 8 developmental stages of three strains of *Ae. aegypti* mosquitoes for transcripts (A) AAEL013584-RA and (B) AAEL10196-RA, (C) AAEL013712-RA , (D) AAEL013713-RA showing the PCR bands classification as N/A, +/-, + or ++ .
