# Supplementary material for: Strain Variation in the Transcriptome of the Dengue Fever Vector, Aedes aegypti
Source: G3 (Bethesda). 2012 Jan 1;2(1):103–14. doi: 10.1534/g3.111.001107 (PMC3276191; doi:10.1534/g3.111.001107)
Supplement: Supporting Information [file supp_2.1.103_FigureS6.pdf]

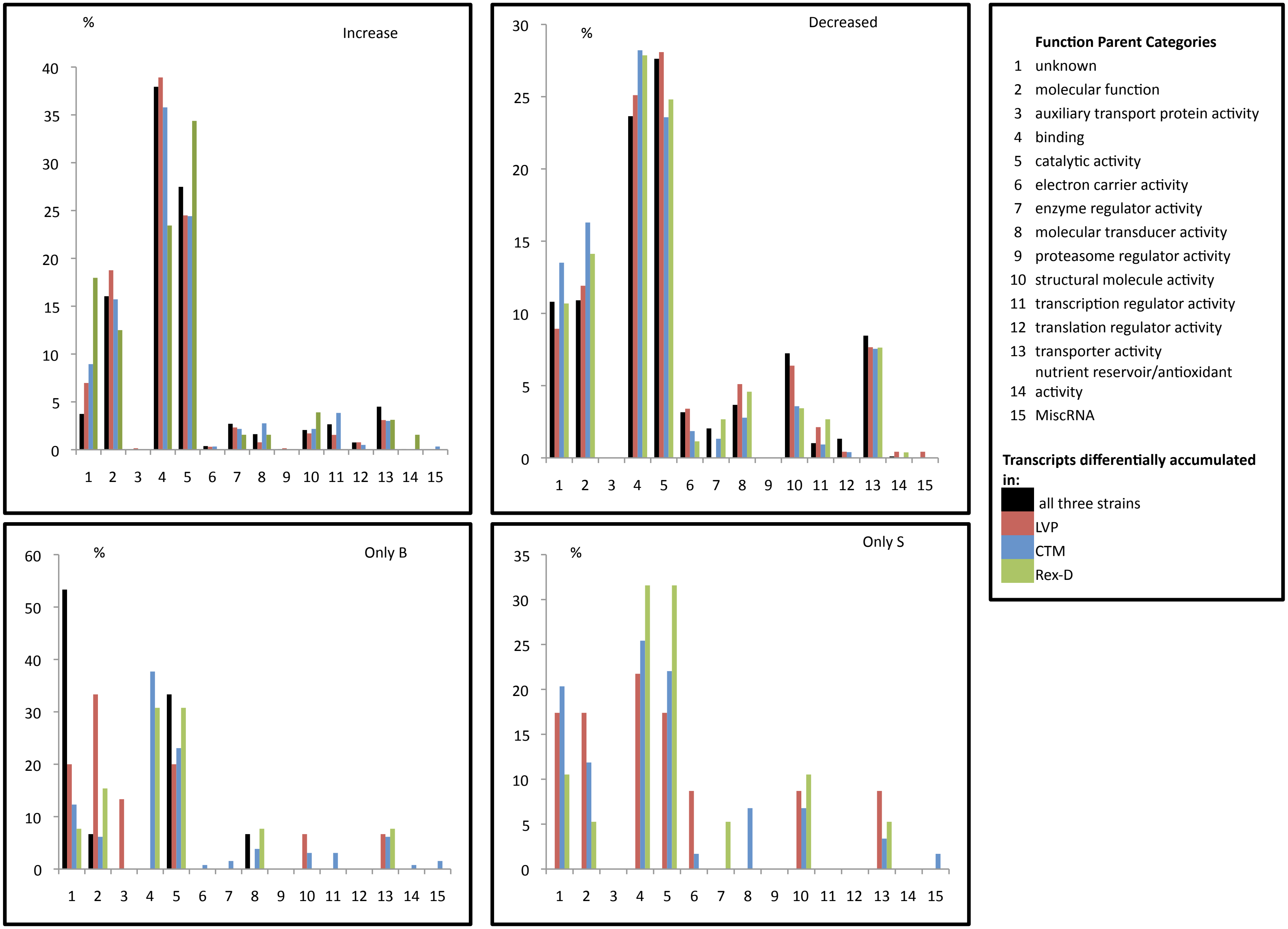

Figure S6 Function parent of differentially-accumulated transcripts. Function parent attribution of the transcripts accumulated differentially at 5 hPBM mosquitoes of the LVP, CTM and Rex-D strains.
