# Supplementary material for: Strain Variation in the Transcriptome of the Dengue Fever Vector, Aedes aegypti
Source: G3 (Bethesda). 2012 Jan 1;2(1):103–14. doi: 10.1534/g3.111.001107 (PMC3276191; doi:10.1534/g3.111.001107)
Supplement: Supporting Information [file supp_2.1.103_FigureS7.pdf]

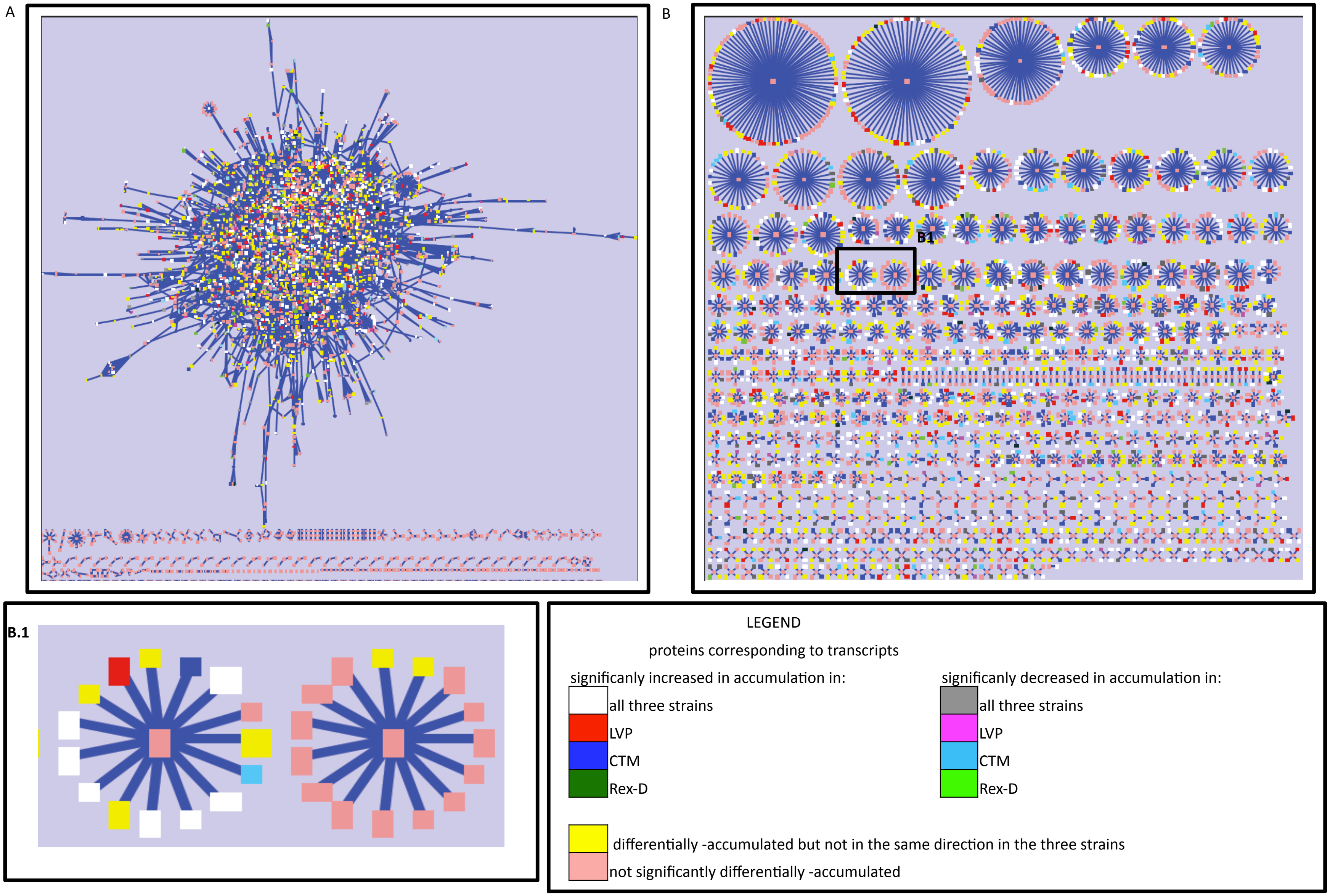

Figure S7 Protein network. *Aedes aegypti* protein network (A) and derived functional modules (B) (Guo *et al*., 2010). Proteins corresponding to transcripts identified by RNA-seq as accumulated differentially between B and S mosquitoes in three *Ae. aegypti* strains analyzed are in different colors. Examples of modules with an enrichment, or exclusive presence ,of proteins corresponding to transcripts either not responsive or differentially accumulated 5hPBM are show in panel B1.
