# Supplementary material for: Strain Variation in the Transcriptome of the Dengue Fever Vector, Aedes aegypti
Source: G3 (Bethesda). 2012 Jan 1;2(1):103–14. doi: 10.1534/g3.111.001107 (PMC3276191; doi:10.1534/g3.111.001107)
Supplement: Supporting Information [file supp_2.1.103_FigureS8.pdf]

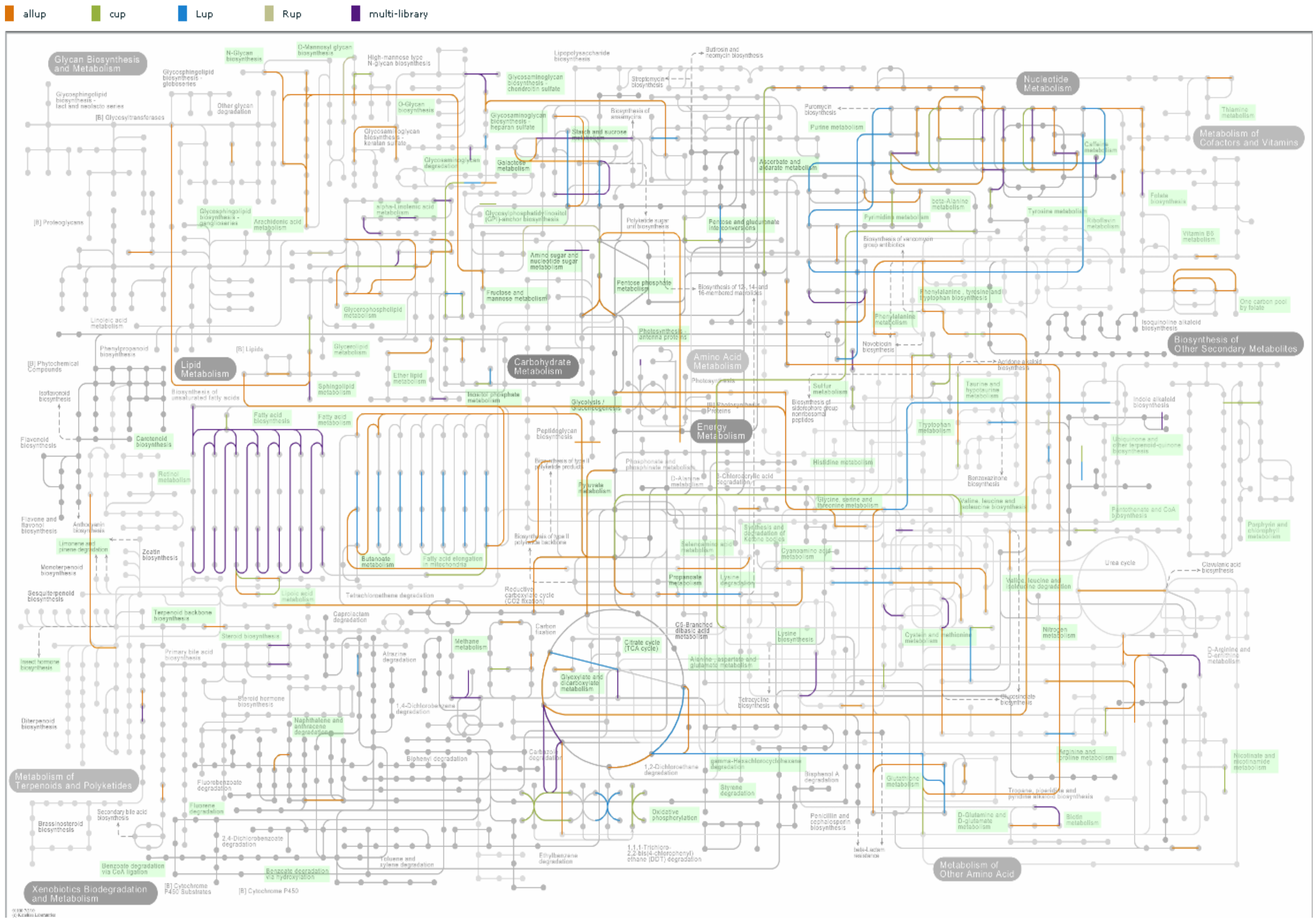


**Figure S8** Metabolic pathways corresponding to transcripts increased in accumulation 5hPBM in all three strains (allup), in LVP (Lup), in CTM (cup) and in Rex-D (Rup) are visualized by LinkinPath (Ingsriswang et al., 2011). Multi-library corresponds to pathways elicited by transcripts increased in accumulation in more than one tested condition.
