# Supplementary material for: Strain Variation in the Transcriptome of the Dengue Fever Vector, Aedes aegypti
Source: G3 (Bethesda). 2012 Jan 1;2(1):103–14. doi: 10.1534/g3.111.001107 (PMC3276191; doi:10.1534/g3.111.001107)
Supplement: Supporting Information [file supp_2.1.103_TableS1.pdf]

Table S1 RT-PCR primers and conditions.

Best match to PFAM database	Transcript ID	Forward	Reverse	TA	ET	N. cyc.
Ins_allergen_rp	AAEL013127-RB	CTACTTAACCACTGACAAGGAATTTC	ATGAGATAGCACAAAAAAATTAGAGGTTA	60C	30 sec	25
	AAEL013584-RA	CATCAGATACATCACAATCGATCAAAGAACC	CCTTCAGGGTGCGTGTGTCTG	68C	30 sec	30
	AAEL013577-RA	TCCATCAGCAAGTAGATCTACAACAC	AAGTCATCCTTCAGGGCACG	62C	30 sec	30
	AAEL013577-RB	TCCATCAGCAAGTAGATCTACAACAC	AAGTCATCCTTCAGGGCACG	62C	30 sec	30
	AAEL010431-RA	TCCATCAGCAAGTAGATCTACAACAC	AAGTCATCCTTCAGGGCACG	62C	30 sec	30
	AAEL010429-RA	TCCATCAGCAAGTAGATCTACAACAC	AAGTCATCCTTCAGGGCACG	62C	30 sec	30
	AAEL009166-RA	TCTGGTCGTACATTACTTCA	GTACACAAAGTCTCACTTAGAAAC	54C	45 sec	25
	AAEL013118-RA	TTTTATGATTTATGTGTACTGACATATTC	TTTTATAACTGGTTTTTAGTATCCCC	57C	30 sec	35
	AAEL001621-RA	GTAACAAATATACGTAATGTTTTAAAACATCAG	GTATGATTTCTAAAAGGTTAACCTTAAACATTT	60C	30 sec	30
Peptidase_C1	AAEL015312-RA	CACAGCACGTAGAGATGTTCC	CCTGGCTCCAAAGTAGGACTGG	60C	30 sec	30
	AAEL012216-RA	TGCACAGCACAAATAACCCCC	CCTGGCTCCAAAGTAGGACTGG	65C	30 sec	30
	AAEL007585-RA	TGCACAGCACAAATAACCCCC	CCTGGCTCCAAAGTAGGACTGG	65C	30 sec	30
Trypsin	AAEL013713-RA	CCACTGACTTCGGCCAC	GGCAATACCCTGGTCGGAGTT	62	30 sec	30
	AAEL013712-RA	CGTTGAGCGGGGTTGGA	CATAGTCGGTGGTAACTTCATCGAT	62	30 sec	30
	AAEL010196-RA	*ACTCTTGCCAGGGTGATTCT	*TATTTTTAATCATTTCATTA	45C	30 sec	30
others	AAEL013706-RA	CCACCGACTACGATTTTGCG	GGAGAACTTCACGATTCAGCTGG	65C	30 sec	30
Vitellogenin_N	AAEL006126-RB	GATCAAGCTTCACCAGTGTCTTACAG	CATACTCTGGCTGCTTGACGTAG	63C	45 sec	30
	AAEL002908-RA	CGGAGACTACTGAGCAGTTATGGG	TCTAATGTAACTTTCAGACATTTTCG	59C	30 sec	35

*from Brackney *et al.*, 2010.

Primer pairs and gene amplification conditions (Annealing temperature=TA, extension time=ET, number of cycles=N.cyc) used to assay the abundance of the listed transcripts during *Ae. aegypti* mosquito development.
