# Supplementary material for: Strain Variation in the Transcriptome of the Dengue Fever Vector, Aedes aegypti
Source: G3 (Bethesda). 2012 Jan 1;2(1):103–14. doi: 10.1534/g3.111.001107 (PMC3276191; doi:10.1534/g3.111.001107)
Supplement: Supporting Information [file supp_2.1.103_TableS6.pdf]

Table S6 Average fold changes of a random selection of thirteen genes in sugar- (S) and blood-fed (B) *Ae. aegypti* mosquitoes of three different strains as detected by qPCR.

		CTM			
transcript-ID	Function descriptors	B (std)	S (std)	average fold changes	t test
AAEL006138-RA	Vitellogenin-A1	1.57 (0.93)	1.4E-3 (3.21E-4)	10.1	t=3.29 (df=11) p=0.007
AAEL013284-RA	Serine-type endopeptidase AaLT	0.44 (0.16)	2.02 E-3 (1.08E-3)	7.75	t=5.44 (df=9) p<0.001
AAEL013707-RA	Trypsin-1	0.59 (0.52)	7.11E-3 (8.26 E-3)	6.39	t=2.58 (df=10) p=0.027
AAEL001806-RA	Lipid binding	0.21 (0.12)	2.84E-2 (2.40 E-3)	2.88	t=2.93 (df=7) p=0.022
AAEL014734-RA	Catalytic activity	0.16 (7.96 E-2)	1.80 E-2 (9.00 E-3)	3.19	t=3.60 (df=7) p=0.009
AAEL011470-RA	Protein binding	6.34E-2 (2.93 E-2)	4.72E-2 (1.82E-2)	0.42	t=1.23 (df=7) p=0.340
AAEL013005-RA	Molecular function	1.69E-2 (5.43E-3)	1.42E-2 (4E-3)	0.42	t=0.81 (df=6) p=0.451
AAEL002565-RA	Structural constituent of Cytoskeleton	7.38E-2(4.70E-2)	0.15(0.10)	-1.03	t=-1.82 (df=11) p=0.096
AAEL008848-RA	Catalytic activity	0.39 (0.24)	0.55 (0.33)	-0.51	t=-1.02 (df=11) p=0.328
AAEL012175-RA	Catalytic activity	0.59 (0.58)	1.16 (0.83)	-0.98	t=-1.52 (df=12) p=0.153
AAEL011871-RA	Electron Transporter	7.6E-2 (0.11)	9.25E-2 (9.17E-2)	6.6	t=-0.27 (df=9) p=0.792
AAEL006425-RA	Trypsin	8.25E-2 (8.18 E-2)	0.83 (0.15)	-3.33	t=-9.59 (df=8) p<0.001
AAEL008701-RA	Iron Ion Binding	1.07E-3 (5.10E-4)	1.90E-2 (6.91E-3)	-4.15	t=-6.39(df=9) p<0.001

		Rex-D			
**transcript-ID**	**Function descriptors**	B (std)	S (std)	average fold changes	t test
AAEL006138-RA	Vitellogenin-A1	2.69 (0.91)	2E-2 (2.95E-2)	7.05	t=6.53 (df=8) p<0.001
AAEL013284-RA	Serine-type endopeptidase AaLT	1.25 (0.36)	2.38E-2 (5.55 E-2)	5.71	t=8.30 (df=9) p<0.001
AAEL013707-RA	Trypsin-1	0.41 (7.50E-2)	1.49 E-2 (9.75 E-3)	4.78	t=10.45 (df=6) p<0.001
AAEL001806-RA	Lipid binding	0.17 (0.04)	2.63E-2 (7.23 E-3)	2.65	t=7.08 (df=7) p<0.001
AAEL014734-RA	Catalytic activity	3.88 E-2 (2.96 E-2)	4.75 E-3 (3.73 E-3)	3.03	t=2.87 (df=8) p=0.021
AAEL011470-RA	Protein binding	2.09 (2.34E-2)	2.13 (0.16)	-0.03	t=-0.60 (df=9) p=0.566
AAEL013005-RA	Molecular function	1.80E-2 (4.29E-3)	7.11E-3(2.26E-4)	1.34	t=5.08 (df=6) p=0.002
AAEL002565-RA	Structural constituent of Cytoskeleton	6.75E-2(1.11E-2)	7.32E-2(2.88E-2)	-0.12	t=-0.37 (df=6) p=0.724
AAEL008848-RA	Catalytic activity	0.21 (0.02)	0.28(1.54)	0.62	t=0.71 (df=8) p=0.503
AAEL012175-RA	Catalytic activity	0.37 (0.25)	0.77 (0.38)	-3.34	t=-2.01 (df=9) p=0.076
AAEL011871-RA	Electron Transporter	0.45 (0.13)	0.69 (0.44)	-0.62	t=-1.19(df=9) p=0.266
AAEL006425-RA	Trypsin	2.84E-2 (4.00E-2)	0.39 (0.17)	-3.78	t=-4.53(df=8) p=0.002
AAEL008701-RA	Iron Ion Binding	1.89E-3 (2.11E-3)	1.91E-2 (5.99E-3)	-3.34	t=-4.22(df=6) p=0.006

Results for LTV are as previously reported (Bonizzoni *et al*., 2011).
