# Supplementary material for: Strain Variation in the Transcriptome of the Dengue Fever Vector, Aedes aegypti
Source: G3 (Bethesda). 2012 Jan 1;2(1):103–14. doi: 10.1534/g3.111.001107 (PMC3276191; doi:10.1534/g3.111.001107)
Supplement: Supporting Information [file supp_2.1.103_TableS8.pdf]

Table S8 Fold-changes between blood- and sugar-fed *Ae. aegypti* mosquitoes of three different strains as detected by qPCR at 5, 8, 12 and 24 hPBM.

			LTV			
transcript ID	Function descriptors		B (std)	S (std)	average fold-changes	
					Actual	Log2
AAEL001806-RA	Lipid binding	5 hPBM	0.14 (3.30E-2)	5.05E-2 (1.36E-2)	2.84	1.50589093
		8 hPBM	0.45 (9.40E-2)	5.05E-2 (1.36E-2)	8.93	3.158660175
		12 hPBM	0.50 (0.11)	5.05E-2 (1.36E-2)	10.01	3.323370069
		24 hPBM	0.27 (5.20E-2)	5.05E-2 (1.36E-2)	5.38	2.427606173
AAEL006138-RA	Vitellogenin-A1	5 hPBM	1.40 (0.86)	7.08e-3 (1.0E-2)	197.84	7.628190335
		8 hPBM	9.44 (2.66)	7.08e-3 (1.0E-2)	1332.6	10.38002808
		12 hPBM	16.49 (3.99)	7.08e-3 (1.0E-2)	2328.64	11.18527191
		24 hPBM	58.39 (21.13)	7.08e-3 (1.0E-2)	8246.53	13.00957147
AAEL013707-RA	Trypsin-1	5 hPBM	3.05 (0.66)	1.75E-2(8.66E-3)	174.52	7.447248569
		8 hPBM	1.59 (1.29)	1.75E-2(8.66E-3)	90.96	6.507160349
		12 hPBM	0.29 (7.04E-2)	1.75E-2(8.66E-3)	13.64	3.769771739
		24 hPBM	4.82E-2 (5.3E-2)	1.75E-2(8.66E-3)	2.75	1.459431619
AAEL006425-RA	Trypsin	5 hPBM	7.65E-2 (2.48E-2)	0.15 (5.20E-2)	0.51	-0.971430848
		8 hPBM	7.52 E-2 (3.0 E-2)	0.15 (5.20E-2)	0.5	-1
		12 hPBM	4.73 E-2 (5.22E-3)	0.15 (5.20E-2)	0.31	-1.689659879
		24 hPBM	0.18 (7.48E-2)	0.15 (5.20E-2)	0.03	-5.058893689
AAEL013284-RA	Serine-type endopeptidase AaLT	5 hPBM	0.49 (0.25)	6.47E-3 (4.18E-3)	75.65	6.241268177
		8 hPBM	4.04 (0.72)	6.47E-3 (4.18E-3)	269.58	8.074569657
		12 hPBM	4.40 (0.72)	6.47E-3 (4.18E-3)	173.47	7.438542374
		24 hPBM	1.72 (1.01)	6.47E-3 (4.18E-3)	2.42E-04	-12.02467797

transcript ID	Function descriptors		CTM			
			B (std)	S (std)	average fold-changes	
					Actual	Log2
AAEL001806-RA	Lipid binding	5 hPBM	0.12 (2.31E-2)	3.41E-2 (9.63 E-3)	3.38	1.757023247
		8 hPBM	0.20 (3.17E-2)	3.41E-2 (9.63 E-3)	5.9	2.560714954
		12 hPBM	0.50 (0.18)	3.41E-2 (9.63 E-3)	14.73	3.880685525
		24 hPBM	6.94E-2 (3.25E-2)	3.41E-2 (9.63 E-3)	2.04	1.028569152
AAEL006138-RA	Vitellogenin-A1	5 hPBM	1.54 (0.76)	1.4E-3 (3.21E-4)	609.14	9.250630034
		8 hPBM	8.00 (1.41)	1.4E-3 (3.21E-4)	3158.14	11.62485941
		12 hPBM	24.87 (7.70)	1.4E-3 (3.21E-4)	9814.12	13.2606432
		24 hPBM	35.48 (5.24)	1.4E-3 (3.21E-4)	13999	13.77303512
AAEL013707-RA	Trypsin-1	5 hPBM	0.72 (0.37)	4.91E-3 (1.15 E-3)	143.32	7.163096138
		8 hPBM	0.33 (8.36E-2)	4.91E-3 (1.15 E-3)	66.29	6.050719348
		12 hPBM	0.179 (5.79E-2)	4.91E-3 (1.15 E-3)	36.42	5.186659017
		24 hPBM	1.13E-2 (7.78E-3)	4.91E-3 (1.15 E-3)	2.3	1.201633861
AAEL006425-RA	Trypsin	5 hPBM	2.67E-2 (1.21E-2)	0.47 (0.13)	0.057	-4.13289427
		8 hPBM	3.82 E-2 (3.89E-2)	0.47 (0.13)	0.08	-3.64385619
		12 hPBM	3.33 E-2 (2.02E-2)	0.47 (0.13)	0.07	-3.836501268
		24 hPBM	6.95E-2 (3.24E-2)	0.47 (0.13)	0.1	-3.321928095
AAEL013284-RA	Serine-type endopeptidase AaLT	5 hPBM	0.28 (0.11)	1.14 E-3 (129E-3)	250.45	7.968378801
		8 hPBM	1.88 (0.54)	1.14 E-3 (129E-3)	1657.58	10.69486279
		12 hPBM	3.95 (1.30)	1.14 E-3 (129E-3)	3479.47	11.76465185
		24 hPBM	2.79 (1.03)	1.14 E-3 (129E-3)	2458.05	11.26329855

transcript ID	Function descriptors		Rex-D			
			B (std)	S (std)	average fold-changes	
					actual	Log2
AAEL001806-RA	Lipid binding	5 hPBM	0.13 (1.36E-2)	3.47E-2 (1.08E-2)	3.88	1.956056652
		8 hPBM	0.18 (4.91E-2)	3.47E-2 (1.08E-2)	5.3	2.40599236
		12 hPBM	0.25 (0.14)	3.47E-2 (1.08E-2)	7.32	2.871843649
		24 hPBM	3.45E-2 (1.09E-2)	3.47E-2 (1.08E-2)	0.99	-0.01449957
AAEL006138-RA	Vitellogenin-A1	5 hPBM	1.53 (0.30)	1.53E-3 (7.98E-4)	1000.65	9.966721732
		8 hPBM	4.33 (1.75)	1.53E-3 (7.98E-4)	2829.58	11.46637221
		12 hPBM	7.44 (0.88)	1.53E-3 (7.98E-4)	4863.71	12.24784149
		24 hPBM	1.53E-3 (8.03E-4)	1.53E-3 (7.98E-4)	1.001	0.001441974
AAEL013707-RA	Trypsin-1	5 hPBM	1.35 (8.72E-2)	2.79 E-3 (5.40E-4)	484.74	8.921067326
		8 hPBM	0.72 (0.21)	2.79 E-3 (5.40E-4)	270.42	8.079058046
		12 hPBM	0.45 (0.26)	2.79 E-3 (5.40E-4)	160.5	7.326429487
		24 hPBM	1.26E-2 (1.12E-2)	2.79 E-3 (5.40E-4)	4.52	2.176322773
AAEL006425-RA	Trypsin	5 hPBM	3.07E-2 (9.94E-3)	0.35 (0.35)	0.09	-3.473931188
		8 hPBM	1.74 E-2 (5.09E-3)	0.35 (0.35)	0.05	-4.321928095
		12 hPBM	8.14E-2 (6.27E-2)	0.35 (0.35)	0.23	-2.120294234
		24 hPBM	0.13 (2.62E-2)	0.35 (0.35)	0.38	-1.395928676
AAEL013284-RA	Serine-type endopeptidase AaLT	5 hPBM	3.06E-4 (1.12E-4)	6.33E-4 (3.43 E-4)	0.48	-1.058893689
		8 hPBM	2.70E-3 (7.85E-4)	6.33E-4 (3.43 E-4)	4.27	2.09423607
		12 hPBM	6.04E-3 (4.44E-3)	6.33E-4 (3.43 E-4)	9.53	3.252476214
		24 hPBM	7.04E-3(5.54E-3)	6.33E-4 (3.43 E-4)	11.11	3.473786912
