# Supplementary material for: Strain Variation in the Transcriptome of the Dengue Fever Vector, Aedes aegypti
Source: G3 (Bethesda). 2012 Jan 1;2(1):103–14. doi: 10.1534/g3.111.001107 (PMC3276191; doi:10.1534/g3.111.001107)
Supplement: Supporting Information [file supp_2.1.103_TableS9.pdf]

Table S9 Expression profile of eighteen *Ae. aegypti* transcripts during development. RT-PCR results of selected transcripts over 8 developmental stages of *Ae. aegypti* mosquitoes. RT-PCR results were classified in four groups as absence of amplification (N/A); faint amplification (+/-); amplification (+); strong amplification (++) (Figure S4).

Best match to PFAM database	transcript-ID (1)	strain	Developmental stages (2)							
Ins_allergen_rp			E	L	P	SF-M	SF-F	BF-5H	BF-24H	BF-72H
	AAEL013127-RB	LTV	N/A	N/A	N/A	N/A	N/A	+	+	N/A
		CTM	N/A	N/A	N/A	N/A	N/A	+	+/-	N/A
		Rex-D (3)	N/A	N/A	N/A	N/A	N/A	N/A	+	N/A
	AAEL013584-RA	LTV	+/-	++	++	+/-	+/-	++	++	+/-
		CTM	N/A	++	++	+/-	+/-	++	++	+/-
		Rex-D	+/-	++	++	+/-	+/-	++	++	+/-
	AAEL013577-RA,AAEL013577-RB,	LTV	N/A	N/A	++	+/-	+/-	++	++	+
	AAEL010431-RA, AAEL010429-RA	CTM	N/A	+/-	++	+	+	++	+/-	+/-
	(4)	Rex-D	+/-	N/A	++	+/-	++	++	++	+/-
	AAEL009166-RA	LTV	N/A	N/A	N/A	N/A	N/A	+	N/A	N/A
		CTM	N/A	N/A	N/A	N/A	N/A	N/A	N/A	N/A
		Rex-D	N/A	N/A	N/A	N/A	N/A	N/A	N/A	N/A
	AAEL013118-RA	LTV	N/A	N/A	N/A	N/A	N/A	+	N/A	N/A
		CTM	N/A	N/A	N/A	N/A	N/A	+	N/A	N/A
		Rex-D	N/A	N/A	N/A	N/A	N/A	+/-	N/A	N/A
	AAEL001621-RA	LTV	N/A	N/A	N/A	N/A	N/A	+	N/A	N/A
		CTM	N/A	N/A	N/A	N/A	N/A	N/A	N/A	N/A
		Rex-D	N/A	N/A	N/A	N/A	N/A	N/A	N/A	N/A
Peptidase_C1	AAEL015312-RA	LTV	+	+	+/-	+/-	+/-	++	++	++
		CTM	+/-	+/-	+	+	+/-	++	++	+
		Rex-D	+	+/-	+/-	+/-	+/-	+	++	+

Best match to PFAM database	transcript-ID (1)	strain	Developmental stages (2)							
	AAEL012216-RA, *AAEL007585-RA*	LTV	+/-	N/A	N/A	N/A	N/A	++	++	++
	(4)	CTM	+/-	+/-	+/-	++	+/-	++	++	++
		Rex-D	+/-	+/-	+/-	+/-	+	++	++	+
Trypsin	*AAEL013713-RA*	LTV	+	+	+/-	+/-	+	++	++	+
		CTM	+	N/A	+	+/-	+	++	++	+/-
		Rex-D	+	+/-	+/-	+/-	+/-	+	+	++
	AAEL013712-RA	LTV	+	+	+	+/-	+	++	++	+
		CTM	+	N/A	+	+/-	+	++	++	+/-
		Rex-D	+	+/-	+	+/-	+	++	+	++
	AAEL0010196-RA	LTV	+/-	+/-	+	+/-	+/-	+	++	+
		CTM	+/-	+/-	+	+/-	+	+	++	+/-
		Rex-D	+/-	+/-	+	+/-	+	+	++	+/-
unknown	AAEL013706-RA	LTV	+	N/A	+	+/-	+/-	++	++	++
		CTM	++	+	++	+	+	++	++	+
		Rex-D	+	+	++	+	+	++	++	+
	AAEL006126-RB	LTV	+	+	++	++	+	++	++	+
		CTM	+	++	++	++	+	++	++	+
		Rex-D	+	+	+	+	+	++	++	+
	AAEL002908-RA	LTV	+/-	+/-	+/-	+/-	+/-	++	++	++
		CTM	+/-	+/-	+/-	+/-	+/-	++	++	++
		Rex-D	+/-	+/-	+/-	+/-	+/-	++	++	++

(1)Transcripts significantly found only in B mosquitoes are in italics, (2) Developmental stages tested are: E=embryos, L=larvae, P=pupae, SF-M=sugar fed males, SF-F=sugar fed females, and blood fed females sampled 5, 24 and 72 hours post blood feeding for BF-5H, BF-24H and BF-72H; (3) when products of the PCR were used as template for a second PCR using forward primer 5' CAAGGAAGTGCTGAACTACTTGGAA and reverse primer 5'GCTTTTAAGCTCTTAACTCCTTTCG, a PCR band of expected size was detected in P, BF-5h, BF-24h; (4) Level of sequence identity among the four transcripts (AAEL013577-RA, AAEL013577-RB, AAEL010431-RA and AAEl010429-RA) and the two transcripts (AAEL012216-RA, AAEL007585-RA) prevented the design of transcript-specific primers.
