# Supplementary material for: Strain Variation in the Transcriptome of the Dengue Fever Vector, Aedes aegypti
Source: G3 (Bethesda). 2012 Jan 1;2(1):103–14. doi: 10.1534/g3.111.001107 (PMC3276191; doi:10.1534/g3.111.001107)
Supplement: Supporting Information [file supp_2.1.103_TableS10.pdf]

Table S10 Comparison of number of transcripts from different functional classes. Fisher exact test for transcripts of different functional classes increased or decreased in accumulation 5 hBPM (A) consistently in all three strains, (B) in LVP, (C) in CTM (D) in Rex-D. Fisher exact test for transcripts of different functional classes (E) increased or (F) decreased in accumulation 5hBPH between LVP and CTM, LVP and Rex-D and CTM and Rex-D.

**A**	N. transcrips		
	Increased	Decreased	Fisher exact test two tailed p value
CS	59	68	<0.0001
DIV	268	75	<0.0001
PT	148	37	<0.0001
DM	9	7	0.4334
TMLCA	275	245	<0.0001
TRP	43	10	0.0186
UNK	265	143	0.6459
TTPMR	384	101	<0.001
STM	97	113	<0.001
**total**	**1548**	**799**	

**B**	N. transcripts		
	Increased	Decreased	Fisher exact test two tailed p value
CS	27	9	0.8495
DIV	77	25	0.4082
PT	50	15	0.388
DM	3	1	0.5816
TMLCA	77	56	0.0002
TRP	11	7	0.3001
UNK	102	31	0.1688
TTPMR	134	23	<0.001
STM	50	42	0.0002
**total**	**531**	**209**	

C

	N. transcripts		
	Increased	Decreased	Fisher exact test two tailed p value
CS	62	44	0.0877
DIV	93	52	0.5196
PT	85	28	0.0488
DM	8	6	0.4072
TMLCA	159	135	<0.001
TRP	28	21	0.1232
UNK	183	100	0.487
TTPMR	281	86	<0.001
STM	135	50	0.0467
**total**	**1034**	**522**	

D

	N. transcripts		
	Increased	Decreased	Fisher exact test two tailed p value
CS	3	16	0.1347
DIV	7	26	0.1732
PT	9	18	1
DM	0	2	1
TMLCA	35	50	0.0424
TRP	3	4	0.6848
UNK	22	31	0.1463
TTPMR	12	34	0.3957
STM	11	34	0.3013
**total**	**102**	**215**	

E

	Fisher exact test two tailed p value		
	LVP/CTM	LVP/Rex-D	CTM/Rex-D
CS	0.4913	0.4524	0.2658
DIV	0.0014	0.0381	0.5838
PT	0.4472	1	0.8501
DM	0.7588	1	1
TMLCA	0.7092	0.0001	0.0001
TRP	0.4974	0.4813	0.7497
UNK	0.4892	0.5869	0.3449
TTPMR	0.432	0.0029	0.0005
STM	0.0385	0.7136	0.6416

**F**	Fisher exact test two tailed p value		
	LVP/CTM	LVP/Rex-D	CTM/Rex-D
CS	0.058	0.2166	0.7673
DIV	0.426	1	0.4295
PT	0.3846	0.7184	0.1332
DM	0.6797	1	1
TMLCA	0.8522	0.4332	0.5132
TRP	0.8318	0.3754	0.1804
UNK	0.2001	1	0.1384
TTPMR	0.0661	0.1568	0.9127
STM	0.002	0.2575	0.0212
