# Supplementary material for: Strain Variation in the Transcriptome of the Dengue Fever Vector, Aedes aegypti
Source: G3 (Bethesda). 2012 Jan 1;2(1):103–14. doi: 10.1534/g3.111.001107 (PMC3276191; doi:10.1534/g3.111.001107)
Supplement: Supporting Information [file supp_2.1.103_TableS12.pdf]

Table S12 Enrichment of immunity-related classes of transcripts. P value associated with a hypergeometric test are shown for mosquitoes of the LVP, CTM and Rex-D strains. p values <0.01 are bold, p values <0.05 are in italicus.

Immunity-related classes	LVP	CTM	Rex-D
1,3-BETA-d Glucan Binding Proteins	0.421205879	0.093204	0.068521
Anti-microbial peptides	0.575258861	0.054398	0.133909
Autophagy Genes	*0.025244527*	*0.032705*	0.10294
Caspase Activator	1	0.636229	1
Caspases	0.596803387	*0.01438*	0.967236
Catalase	0.10875457	0.157481	0.434227
CLIP-domain serine protease	0.665377639	0.624128	0.068302
C-Type Lectins	0.939621148	0.961407	0.773125
Fibrinogen related protein (FREP)	0.729579204	0.963194	0.325004
Galectins	0.596803387	0.767986	0.34444
IMD Pathway members	0.799110015	0.678041	0.627499
inhibitors of apoptosis (IAP)	*0.040252257*	0.259983	*0.047204*
JAKSTAT Signal transduction	0.698987823	0.062487	0.153783
lysozyme	0.421205879	0.573253	0.239038
MD2-like proteins (ML)	0.976981595	0.730233	0.561666
others	*0.016960761*	0.039506	0.165959
peptidoglycan recognition protein	0.139416573	0.095835	0.161615
Peroxidase	*0.01692638*	*0.011764*	**0.000254**
prophenoloxidase	0.979316144	0.971402	0.758062
Relish-like proteins	0.254545294	0.347469	0.153783
Scavanger Receptors	0.086148092	**0.000283**	**0.000854**
Serine protease inhibitors	0.552011966	0.090862	0.100341
Small Regulatory RNA pathway members	**0.000923729**	*0.011925*	*0.033746*
spaetzle like (SPZ like)	0.851962443	0.095835	0.694443
Superoxido-dismutase	0.523084665	0.678041	0.110766
Thio-Ester containing proteins (TEP)	0.167866594	0.837272	0.239038
Toll Receptors/Pathway	0.22889149	0.46441	0.24858
