# Supplementary material for: Strain Variation in the Transcriptome of the Dengue Fever Vector, Aedes aegypti
Source: G3 (Bethesda). 2012 Jan 1;2(1):103–14. doi: 10.1534/g3.111.001107 (PMC3276191; doi:10.1534/g3.111.001107)
Supplement: Supporting Information [file supp_2.1.103_FigureS5.pdf]

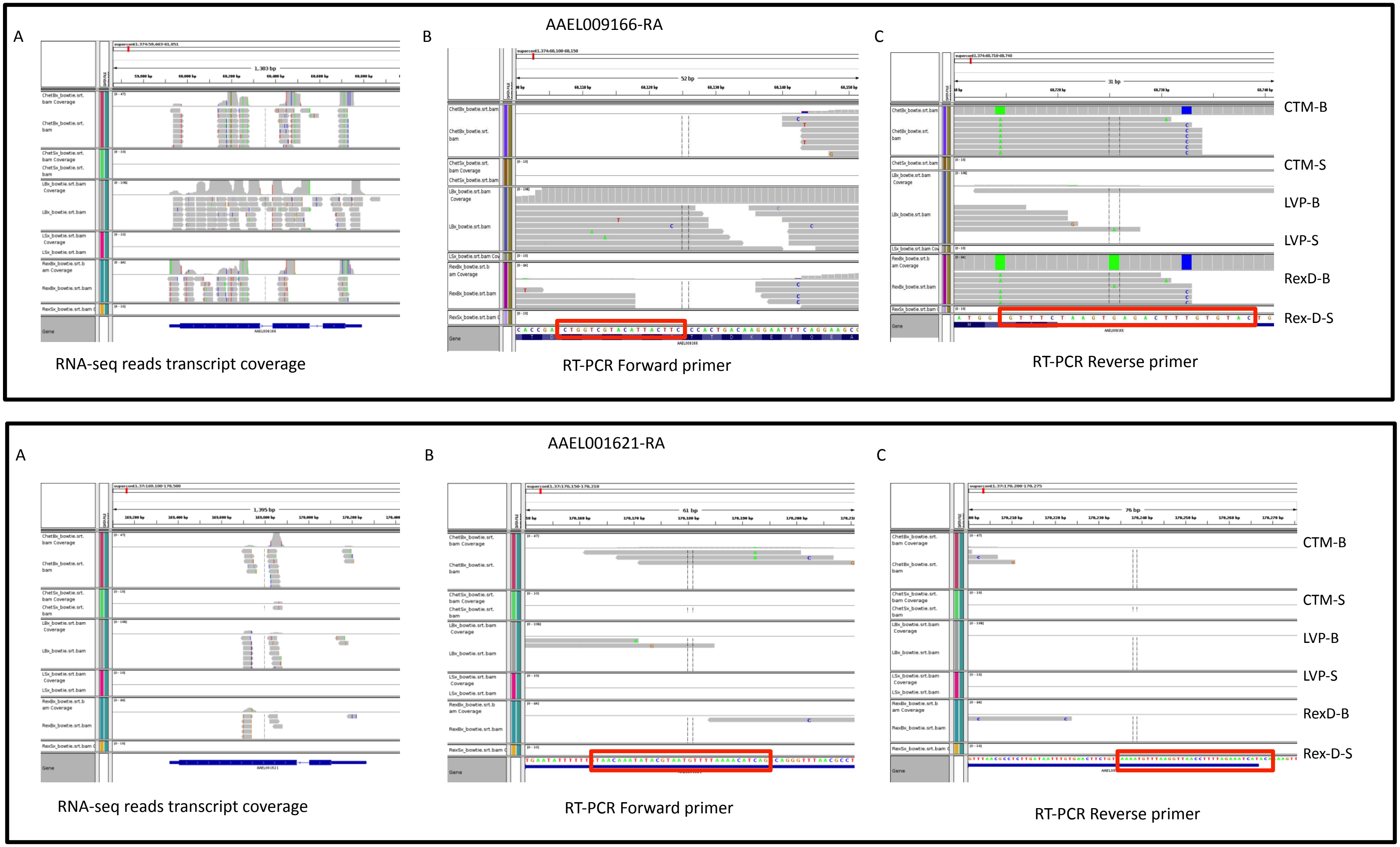

Figure S5 RNA-seq reads coverage (A) and RT-PCR primers position (B,C) in the three *Ae. aegytpi* strain tested for transcript AAEL009166-RA and AAEL001621-RA. In each read (gray raw), mismatches with respect to the reference *Ae. aegypti* genome sequence are shown in colors within the gray raw. RNA-seq reads were mapped to the *Ae. aegypti* genome reference sequence requiring unique match with a maximum of two mismatches. As a consequence, the absence of coverage along a transcript can be interpreted as not-unique sequence within the *Ae. aegypti* genome and/or sequence with high variation (i.e. more than 2 mismatches, insertions, deletions).
